# Three new species of *Trigonospila* Pokorny (Diptera: Tachinidae), from Area de Conservación Guanacaste, northwestern Costa Rica, with a key for their identification

**DOI:** 10.3897/BDJ.3.e4595

**Published:** 2015-08-11

**Authors:** AJ Fleming, D. Monty Wood, Daniel H Janzen, Winnie Hallwachs, M. Alex Smith

**Affiliations:** ‡ Agriculture Agri-Food Canada, Ottawa, Canada; § University of Pennsylvania, Philadelphia, PA, United States of America; | Department of Integrative Biology, Guelph, Canada

**Keywords:** *
Trigonospila
*, Diptera, Tachinidae, tropical rain forest, tropical dry forest, parasitoid fly, host-specificity, caterpillars

## Abstract

We describe three new species of *Trigonospila* Pokorny (Tachinidae: Blondeliini) from Area de Conservación Guanacaste (ACG), northwestern Costa Rica. All were reared from ­various species of ACG caterpillars during an ongoing inventory of caterpillars, their food plants and their parasitoids in dry forest, rain forest and cloud forest. By coupling morphology, photographic documentation, life history and molecular data, we provide a clear and concise description of each species. All species published as new, are known to be previously undescribed as a result of careful study of the genus by DMW. This study builds on the current knowledge of the genus by adding three new species to the current 7 described in the New World. *Trigonospila edwinbermudezi* **sp. n.**, *Trigonospila uniformis* **sp. n.**, and *Trigonospila josemariamoragai* **sp. n.** are all authored and described as new by Fleming and Wood, with a key to their identification. The authors also offer a new record and description of the previously unknown male of *Trigonospila panamensis* (Townsend), reared from ACG caterpillars.

## Introduction

The tachinid genus *Trigonospila* Pokorny 1886, is a small cosmopolitan genus in the tribe Blondeliini of the Exoristinae (Diptera, Tachinidae) (Wood 1985, O'Hara and Wood 2004). The blondeliines are a polyphyletic group (Wood 1985) widely distributed throughout the New World, making up approximately 10% of the tachinid fauna in North America. The concept of the tribe Blondeliini was originally proposed by Mesnil (1939) and has remained largely unchanged to date. Wood (1985) provided a concise diagnosis of the tribe based on Mesnil's work: 1) prosternum setose (though this character has been noted to be variable within *Trigonospila*), 2) first postsutural supraalar bristle, smaller than first postsutural dorsocentral, 3) bend of wing vein M, rounded and obtuse angled, 4) subapical scutellar bristles, long stout and divergent (Wood suggested this character may be variable with respect to tribal placement), and 5) wing vein R_4+5_ ending at or near the wing margin (Wood 1985).

The genus *Trigonospila* is widely distributed, occurring throughout the Old World, in Eurasia, Australia, Oriental and in the Afrotropical regions (Crosskey 1973, Crosskey 1980, O'Hara 2014), and seven species included in the New World, three species occurring north of Mexico, and four in South America (Wood and Zumbado 2010). The genus was originally described from a male specimen, under the type species *Trigonospila picta*, collected in southeastern Styria in what was then referred to as 'bundesland'. Some authors have interpreted Pokorny's original text as having the specimen originating from "monte Wechsel", but this was an erroneous translation as this is referring to a mountain pass, and not a proper noun. It was originally characterized as a small gray and black fly with an elongate cone-shaped abdomen, adorned with silver or gold tomentose bands of varying thickness. Males and females are dimorphic, with only females possessing two proclinate fronto-orbital bristles, apically pointed and downwardly incurved abdomen, as well as significant differences in coloration and pattern. Host preference in *Trigonospila* has been poorly understood, to date there have been no rearing records for any of the New World species with only few specimens being represented in collections. Crosskey (1973) recorded two lepidopterous hosts, belonging to Oecophoridae and Gelechiidae, for an Australian species, *T. brevifacies* (Hardy). Shima (2006) reported the emergence of *T. vittigera* from a coleopteran host in the family Tenebrionidae. Soto and Ocampo (2011), also suggested the emergence of an alleged species of *Trigonospila* from a coleopteran host in the family Curculionidae, however examination of these specimens by both DMW and AJF was not consistent with a diagnosis of *Trigonospila* (Unpublished data).

Herein we describe three new species of the genus *Trigonospila*, and describe the male of *T. panamensis* (Townsend), all reared from wild-caught caterpillars collected from Area de Conservación Guanacaste, in northwestern Costa Rica. The decision that these three are previously undescribed is based on examination of New World *Trigonospila*, and observation of differences in external morphology, and CO1 gene sequences. By coupling CO1 data with morphological descriptions we are able to show that the coloration patters of the abdomens are not only differences between males and females but they are consistent within species making them useful in visual species identification. This paper adds to the existing knowledge of *Trigonospila* by providing new records relating to distribution and host preference.

## Materials and methods

As this paper forms part of a larger series dedicated to naming the tachinid fauna of ACG, the methods described herein are in referenced and adapted from earlier works by the authors (Fleming et al. 2014a, Fleming et al. 2014b, Fleming et al. 2015).

### Acronyms for depositories.

BMNH - The Natural History Museum, London, United Kingdom

CNC - Canadian National Collection of Insects, Arachnids and Nematodes, Ottawa, Canada

USNM - U.S. National Museum of Natural History, Washington, D.C., USA

INBIO - ​Instituto Nacional de Biodiversidad, Santo Domingo de Heredia, Costa Rica

### Geographic area of the study and rearing intensity

All flies and rearing information described here were found by the 35+ year–old ongoing inventory of the caterpillars, their food plants and their parasitoids of the dry forest, rain forest, cloud forest, and intergrades, in the 125,000+ ha terrestrial portion of Area de Conservación Guanacaste (ACG) in northwestern Costa Rica (Fernandez-Triana et al. 2014, Janzen and Hallwachs 2011, Janzen et al. 2009, Janzen et al. 2011, Rodriguez et al. 2013, Smith et al. 2006, Smith et al. 2007, Smith et al. 2008, Smith et al. 2009, Smith et al. 2012). The parasitoid rearing methods are described at http://janzen.bio.upenn.edu/caterpillars/methodology/how/parasitoid_husbandry.htm. In brief, caterpillars (and sometimes pupae) are found in the wild at all instars by a wide variety of search methods, and reared in captivity on the food plant species on which they were found, until they produce an adult, a parasitoid, or die of other causes. Each caterpillar is documented as an individual, as are the adult parasitic flies. The caterpillars used in this study are a result of the tireless efforts of ACG staff and collaborators. Through these efforts, science has garnered an unprecedented amount of data, providing invaluable information on parasitoid biology and that of the associated hosts.

This inventory has reared about 600,000 wild-caught caterpillars since 1978. All frequencies of parasitism reported here need to be considered against this background inventory. Equally, it is patently obvious that the inventory searches some kinds of vegetation and height off the ground much more thoroughly than others, and it also searches throughout the year. Comparison of reared species of parasitoids with those collected by net or Malaise traps demonstrates that to date, the caterpillar inventory has so far encountered well less than half the species of caterpillar parasitoids present in ACG. The largest unsampled void is the upper foliage of the canopy above about 3–4 m above the ground.

The treatment reported here is focused on placing names on the species reared, thereby preparing them for later detailed ecological and behavioral accounts and studies that will normally extend across ACG ecological groups, whole ecosystems, and taxonomic assemblages much larger than a genus.

### Imaging

Our descriptions of new species are deliberately brief and only include some differentiating descriptions of body parts and colors that are commonly used in tachinid identification. These brief descriptions are complemented with an extensive series of color photos of every species to illustrate the readily-observed differences among them.

Habitus photographs were taken using a Canon T3i digital SLR, equipped with a 65mm Macro Photo Lens 1:2.8 (MP–E 65mm), mounted on a microscope track stand (AmScope, Model: TS200) modified to accept a Manfrotto QR 200PL–14 quick release plate. Images were shot in aperture priority, allowing the camera to control shutter speed at f/4.5 and take 40 images at equal distance increments. Illumination was provided with a homemade reflective dome (instruction for dome creation can be found at: http://www.cdfa.ca.gov/plant/ppd/entomology/Dome/kd-200.html) placed over a 144 LED ringlight (AmScope, Model: LED–144–YK).

The photographic series was processed from RAW format using Photoshop CS6, and digitally stacked. Each final composite image was created using Zerene Stacker Software v1.04 maximizing image quality and depth of field.

All specimens listed as examined are considered paratypes, except for the holotype which is noted separately.

Wherever a specimen label has been examined, the information is presented using the following symbols: /, indicates the end of a line; //, indicates the end of a label. Labels are presented from top (closest to the specimen) to bottom, with any comments about the label being given in square brackets.

### Voucher specimen management

All caterpillars reared from the ACG efforts receive a unique voucher code in the format of yy–SRNP–xxxxx. Any parasitoid emerging from this caterpillar receives the same voucher code, and then if/when later the parasitoid is dealt with individually, it receives a second voucher code unique to it, in the format of DHJPARxxxxxxx. The voucher codes and collateral data assigned to both host and emergent parasitoids are available at http://janzen.bio.upenn.edu/caterpillars/database.lasso. To date, all DHJPARxxxxxxx coded tachinids have had one leg removed for attempted DNA barcoding at the Biodiversity Institute of Ontario (BIO) in the University of Guelph, with all collateral data and all successful barcodes permanently and publically deposited in the Barcode of Life Data System (BOLD, http://www.boldsystems.org) (Ratnasingham and Hebert 2007), and later migrated to GenBank as well. A neighbor–joining (NJ) tree (Saitou and Nei 1987) for all *Trigonospila* reared and DNA barcoded by this inventory through 2013 is included as a Suppl. material 1. The inventory grows continually and new specimens can be found by searching the genus *Trigonospila* in BOLD. Each barcoded specimen also has an accession code in the Barcode of Life Data System (BOLD) and GenBank.

Inventoried Tachinidae were collected under Costa Rican government research permits issued to DHJ since 1978, and likewise exported under permit by DHJ from Costa Rica to Philadelphia, and then to the final depository in the Canadian National Insect collection in Ottawa, Canada. Tachinid identifications for the inventory were done by DHJ in coordination with a) visual inspection by AJF and DMW, b) DNA barcoding by BIO, MAS, and BOLD, and c) correlation with host caterpillar identifications by DHJ and WH through the inventory itself. Dates of capture of each reared fly in the inventory are the dates of eclosion of the fly, and not the date of capture of the caterpillar. This is because the fly eclosion date is much more representative of the time when that fly species is on the wing than is the time of capture of the caterpillar or (rarely) finding a parasitized pupa. However, the collector listed is the parataxonomist who found the caterpillar, rather than the person who retrieved the newly eclosed fly from its rearing bag or bottle, and processed it by freezing, pinning, labeling and oven–drying. Fly biology and degrees of parasitization by these flies will be the detailed subject of later papers.

### DNA barcoding

DNA barcodes (the standard 5’ region of the mitochondrial cytochrome c oxidase I (COI) gene) for all ACG inventory specimens were obtained using DNA extractions made from single legs using a glass fiber protocol (Ivanova et al. 2006). Total genomic DNA was re-suspended in 30 μl of dH2O, and a 658-bp region near the 5’ terminus of the COI gene was amplified using standard primers (LepF1–LepR1) following established protocols (Smith et al. 2007, Smith et al. 2006, Smith et al. 2008). All information for the sequences associated with each individual specimen (including GenBank and BOLD accession) can be retrieved from the Barcode of Life Data System (BOLD) (Ratnasingham and Hebert 2007) via the publicly available dataset: http://dx.doi.org/10.5883/DS-ASTRIOGO.

### Generic synonyms of *Trigonospila* Pokorny

*Trigonospila* Pokorny 1886: 191. Type species: *Trigonospila picta* Pokorny 1886 (=*Tachina ludio* Zetterstedt 1849), by monotypy.

*Zosteromyia* Brauer and Bergenstamm 1891: 376 (72). Type-species: *Myobia cingulata* Macquart *sensu* Brauer and Bergenstamm (misidentification) (=*Zosteromyia braueri* Townsend 1933), by original designation. Townsend's belief that this species was misidentified needs confirmation (Crosskey 1976). [Type material not examined].

*Succingulum* Pandellé 1894: 52 [no included species]; Pandellé 1896: 148. Type species: *Succingulum transvittatum* Pandellé 1896, by subsequent monotypy. [Type material not examined].

*Panacemyia* Townsend 1919: 164. Type species: *Panacemyia panamensis* Townsend 1919, by original designation. Synonymy by Wood 1985: 85.

*Gymnamedoria* Townsend 1927: 283. Type species: *Gymnamedoria medinoides* Townsend 1927 [=*Succingulum transvittatum* Pandellé 1896, by original designation]. [Type material not examined].

*Zosteromyiopsis* Townsend 1933: 456. Type species: *Myobia cingulata* Macquart 1851, by original designation. [Type material not examined].

*Nimiocauda* Reinhard 1943: 78. Type species: *Nimiocauda erilis* Reinhard 1943, by original designation. Synonymy by Thompson 1963. Wood (1985) was in error in citing *Nimiocauda* as a new synonym, as noted by O'Hara and Wood 2004: 108.

### New World species previously included in *Trigonospila* Pokorny

In the process of species determination, specimens provided from ACG were examined in comparison to the entire known seven-member fauna of the New World *Trigonospila* by both AJF and DMW. These comparisons were made based on geographical proximity of the species, as well as any similarities in life history and morphology. It was found only one species reared in ACG matches any of the known species, *Trigonospila panamensis* (Townsend). Differentiating comparisons are discussed in the descriptions, when necessary. Wherever possible, holotypes were compared to ACG specimens. However, it should be noted if holotype material was unavailable, direct comparisons were made with specimens present at the CNC.

*erilis* Reinhard 1943: 79 (*Nimiocauda*). Holotype female (CNC) [examined by DMW & AJF]. Type locality: United States, New York, Long Island. Type label: Holotype #F: “Wading River/L.I., N.Y./VII-1924//HOLOTYPE/*Nimiocauda*/*erilis*/Reinhard//*Nimiocauda*/*erilis*/R Rnh.//*Panacemyia*/*erilis*/R. Rnh”

*melaleuca* Wulp 1890: 145 (*Hypostena*). Holotype male (BMNH) [examined by DMW]. Type locality Mexico, Tabasco, Teapa. Type label: Holotype #M: “HOLOTYPE//small label with male symbol//Teapa,/Tabasco./March. H.H.S.//B.C.A. Dipt. II./*Hypostena*/*melaleuca*/v.d.W.//Central America./Pres. by/F.D. Godman/O. Salvin/1903-172.//HOLOTYPE/of *Hypostena*/*melaleuca* Wulp/examined 1979/D.M. Wood”

*pallipes* Reinhard 1953: 246 (*Panacemyia*). Holotype female (CNC) [examined by DMW & AJF]. Type locality: United States, Texas, College Station. Type label: Holotype #F: “College Station/May 16, 1946 Tex//H J Reinhard/Collector//HOLOTYPE/*Panacemyia*/*pallipes*/Reinhard//*Panacemyia pallipes*/ R. Rnh”

*panamensis* Townsend 1919: 164 (*Panacemyia*). Holotype male (USNM) [examined by DMW]. Type locality: Panama, Taboga Island. Type label: Holotype #M: “Taboga I Panama/ 26 Febr. 1912/ ABusck coll// Type No./22066 U.S.N.M.//*Panacemyia panamensis*”

*solitaria* Curran 1926: 107 (*Hypostena*). Holotype male (BMNH) [examined by DMW]. Type locality: Jamaica, [Panama], Blue Castle. Type label: Holotype #M: “HOLOTYPE//TYPE/*Hypostena*/(*Tachinophyto*)/*solitaria*/Curran/No.//C.C. GOWDEY//Blue Castle,/ JAMAICA/6.vi.1923/NO.820//Pres. by/Imp. Bur. Ent./Brit. Mus./1927-158.”

*trinitatis* Thompson 1963: 459 (*Panacemyia*). Lectotype female by designation of Wood 1985: 86 (CNC) [examined by DMW & AJF]. Lectotype locality: Trinidad, St. Augustine, ICTA. Type label: Lectotype #F: “ICTA/St Augustine/Trinidad/Aug 1959/Ex Coll. Off Samanea Flowers//F.D. Bennett Collector//LECTOTYPE #f/of *Panacemyia*/*trinitatis* Thompson/ designated 1984/D.M. Wood”

*verticalis* Reinhard 1953: 247 (*Panacemyia*). Holotype male (CNC) [examined by DMW & AJF]. Type locality: United States, Ohio, Amherst. Type label: Holotype #M: “Amherst, Ohio/VI-15 1924//H J Reinhard/Collector//HOLOTYPE/*Panacemyia*/*verticalis*/Reinhard//*Panacemyia*/*verticalis*/R. Rnh”

### Diagnosis of the genus *Trigonospila* Pokorny

Wood (1985) provided a diagnosis and review of the genus which is adapted here. The specimens reared from ACG presented in this study conform to the keys found in Wood (1985) and Wood and Zumbado (2010).

Head: male lacking proclinate fronto-orbital bristles; reclinate orbital bristles of male indistinguishable from frontals; ocellar seta hair-like, nearly parallel to each other in male, parallel or divergent in female; eye bare, or with minute inconspicuous hairs; parafacial bare, extremely narrow; lower margin of face at level of vibrissa not visible in profile; facial ridge with a few small recumbent setae on lower third or less; subvibrissal ridge short, usually with 3 or fewer bristles; anterior margin of postgena concave anteriorly, sloping anteroventrally toward vibrissal angle, without genal dilation; first flagellomere of male about as long as that of female; arista minutely to short pubescent, thickened on basal fourth to fifth.

Thorax: prosternum bare (this character state, a rarity in Blondeliini, is contrary to Mesnil 1939, diagnosis of the tribe, however as with most biological systems, the tribe is not defined by a single character state, but rather the combination of the other diagnostic characters has maintained its present placement; proepisternum bare; postpronotum usually with 2 bristles, or if 3, the inner basal bristle is usually small forming a slightly curved row; katepisternum with 3 bristles, the anteroventral sometimes small; lateral scutellar bristles shorter than subapical bristles, curved medially; apical scutellar bristles usually lacking; fore tibia with 1 posterior bristle; mid tibia with 1 anterodorsal bristle; vein R_2+3_, usually with a single bristle at base of R_4+5_.

Abdomen (Figs 1, 2): abdominal mid-dorsal depression not extending to hind margin of syntergite (ST) 1+2; ST1+2, T3 and T4, each bearing median marginal bristles; median discal bristles almost as long as marginal bristles of same segment; in some species male an extra pair of discal bristles present in front of main pair; abdomen of female strongly curved ventrally.

According to Wood (1985) members of *Trigonospila* share many similarities with *Dolichotarsus* Mesnil, 1977, *Embiomyia* Aldrich, 1934, *Steleoneura* Stein, 1984, and *Pararondania* Villeneuve, 1916. The genus is distinguished from *Steleoneura* by the antennal structure, in which the pedicel of *Steleoneura* is longer than the first flagellomere, and the scapes are separate at their bases; *Steleoneura* also possessing a single relatively long bristle at base of R_4+5_, a single long straight bristle on prosternum, and a small bristle on katepimeron. In *Dolichotarsus* the female abdomen is more laterally compressed, and the membranous ovipositor is fully withdrawn into the abdomen. In the New World species of *Trigonospila*, the arista is less noticeably pubescent than in most of the Old World species, but the female abdomen appears to be similar in all of them.

## Taxon treatments

### Trigonospila josemariamoragai

Fleming & Wood sp. n.

urn:lsid:zoobank.org:act:20CFE09B-9222-4FAB-A9D1-68C74A5750D8

#### Materials

- **Type status:** Holotype. **Occurrence:** occurrenceDetails: http://janzen.sas.upenn.edu; catalogNumber: DHJPAR0042272; recordedBy: D.H. Janzen & W. Hallwachs; individualID: DHJPAR0042272; individualCount: 1; sex: M; lifeStage: adult; preparations: pinned; otherCatalogNumbers: 11-SRNP-70422; **Taxon:** scientificName: Trigonospila josemariamoragai; phylum: Arthropoda; class: Insecta; order: Diptera; family: Tachinidae; genus: Trigonospila; specificEpithet: josemariamoragai; scientificNameAuthorship: Fleming & Wood, 2015; **Location:** continent: Central America; country: Costa Rica; countryCode: CR; stateProvince: Guanacaste; county: Area de Conservacion Guanacaste; locality: Sector Pitilla; verbatimLocality: Sendero Navarro; **Identification:** identifiedBy: AJ Fleming; dateIdentified: 2015; **Event:** samplingProtocol: reared from caterpillar of Egchiretes Poole01 (Nolidae); verbatimEventDate: 21-Mar-2011; **Record Level:** language: en; institutionCode: CNC; collectionCode: Insects; basisOfRecord: Pinned Specimen
- **Type status:** Paratype. **Occurrence:** occurrenceDetails: http://janzen.sas.upenn.edu; catalogNumber: DHJPAR0040982; recordedBy: D.H. Janzen & W. Hallwachs; individualID: DHJPAR0040982; individualCount: 1; sex: M; lifeStage: adult; preparations: pinned; otherCatalogNumbers: 10-SRNP-22521; **Taxon:** scientificName: Trigonospila josemariamoragai; phylum: Arthropoda; class: Insecta; order: Diptera; family: Tachinidae; genus: Trigonospila; specificEpithet: josemariamoragai; scientificNameAuthorship: Fleming & Wood, 2015; **Location:** continent: Central America; country: Costa Rica; countryCode: CR; stateProvince: Guanacaste; county: Area de Conservacion Guanacaste; locality: Sector Del Oro; verbatimLocality: Quebrada Salazar; verbatimElevation: 560; verbatimLatitude: 11.002; verbatimLongitude: -85.463; verbatimCoordinateSystem: Decimal; decimalLatitude: 11.002; decimalLongitude: -85.463; **Identification:** identifiedBy: AJ Fleming; dateIdentified: 2015; **Event:** samplingProtocol: reared from caterpillar of Stenoma exarata (Elachistidae); verbatimEventDate: 03-Jan-2011; **Record Level:** language: en; institutionCode: CNC; collectionCode: Insects; basisOfRecord: Pinned Specimen
- **Type status:** Paratype. **Occurrence:** occurrenceDetails: http://janzen.sas.upenn.edu; catalogNumber: DHJPAR0042259; recordedBy: D.H. Janzen & W. Hallwachs; individualID: DHJPAR0042259; individualCount: 1; sex: F; lifeStage: adult; preparations: pinned; otherCatalogNumbers: 11-SRNP-70435; **Taxon:** scientificName: Trigonospila josemariamoragai; phylum: Arthropoda; class: Insecta; order: Diptera; family: Tachinidae; genus: Trigonospila; specificEpithet: josemariamoragai; scientificNameAuthorship: Fleming & Wood, 2015; **Location:** continent: Central America; country: Costa Rica; countryCode: CR; stateProvince: Guanacaste; county: Area de Conservacion Guanacaste; locality: Sector Pitilla; verbatimLocality: Sendero Navarro; **Identification:** identifiedBy: AJ Fleming; dateIdentified: 2015; **Event:** samplingProtocol: reared from caterpillar of Egchiretes Poole01 (Nolidae); verbatimEventDate: 25-Mar-2011; **Record Level:** language: en; institutionCode: CNC; collectionCode: Insects; basisOfRecord: Pinned Specimen
- **Type status:** Paratype. **Occurrence:** occurrenceDetails: http://janzen.sas.upenn.edu; catalogNumber: DHJPAR0042260; recordedBy: D.H. Janzen & W. Hallwachs; individualID: DHJPAR0042260; individualCount: 1; sex: F; lifeStage: adult; preparations: pinned; otherCatalogNumbers: 11-SRNP-70449; **Taxon:** scientificName: Trigonospila josemariamoragai; phylum: Arthropoda; class: Insecta; order: Diptera; family: Tachinidae; genus: Trigonospila; specificEpithet: josemariamoragai; scientificNameAuthorship: Fleming & Wood, 2015; **Location:** continent: Central America; country: Costa Rica; countryCode: CR; stateProvince: Guanacaste; county: Area de Conservacion Guanacaste; locality: Sector Pitilla; verbatimLocality: Sendero Navarro; **Identification:** identifiedBy: AJ Fleming; dateIdentified: 2015; **Event:** samplingProtocol: reared from caterpillar of Egchiretes Poole01 (Nolidae); verbatimEventDate: 22-Mar-2011; **Record Level:** language: en; institutionCode: CNC; collectionCode: Insects; basisOfRecord: Pinned Specimen
- **Type status:** Paratype. **Occurrence:** occurrenceDetails: http://janzen.sas.upenn.edu; catalogNumber: DHJPAR0042261; recordedBy: D.H. Janzen & W. Hallwachs; individualID: DHJPAR0042261; individualCount: 1; sex: M; lifeStage: adult; preparations: pinned; otherCatalogNumbers: 11-SRNP-70451; **Taxon:** scientificName: Trigonospila josemariamoragai; phylum: Arthropoda; class: Insecta; order: Diptera; family: Tachinidae; genus: Trigonospila; specificEpithet: josemariamoragai; scientificNameAuthorship: Fleming & Wood, 2015; **Location:** continent: Central America; country: Costa Rica; countryCode: CR; stateProvince: Guanacaste; county: Area de Conservacion Guanacaste; locality: Sector Pitilla; verbatimLocality: Sendero Navarro; **Identification:** identifiedBy: AJ Fleming; dateIdentified: 2015; **Event:** samplingProtocol: reared from caterpillar of Egchiretes Poole01 (Nolidae); verbatimEventDate: 22-Mar-2011; **Record Level:** language: en; institutionCode: CNC; collectionCode: Insects; basisOfRecord: Pinned Specimen
- **Type status:** Paratype. **Occurrence:** occurrenceDetails: http://janzen.sas.upenn.edu; catalogNumber: DHJPAR0042262; recordedBy: D.H. Janzen & W. Hallwachs; individualID: DHJPAR0042262; individualCount: 1; sex: F; lifeStage: adult; preparations: pinned; otherCatalogNumbers: 11-SRNP-70440; **Taxon:** scientificName: Trigonospila josemariamoragai; phylum: Arthropoda; class: Insecta; order: Diptera; family: Tachinidae; genus: Trigonospila; specificEpithet: josemariamoragai; scientificNameAuthorship: Fleming & Wood, 2015; **Location:** continent: Central America; country: Costa Rica; countryCode: CR; stateProvince: Guanacaste; county: Area de Conservacion Guanacaste; locality: Sector Pitilla; verbatimLocality: Sendero Navarro; **Identification:** identifiedBy: AJ Fleming; dateIdentified: 2015; **Event:** samplingProtocol: reared from caterpillar of Egchiretes Poole01 (Nolidae); verbatimEventDate: 22-Mar-2011; **Record Level:** language: en; institutionCode: CNC; collectionCode: Insects; basisOfRecord: Pinned Specimen
- **Type status:** Paratype. **Occurrence:** occurrenceDetails: http://janzen.sas.upenn.edu; catalogNumber: DHJPAR0042263; recordedBy: D.H. Janzen & W. Hallwachs; individualID: DHJPAR0042263; individualCount: 1; sex: M; lifeStage: adult; preparations: pinned; otherCatalogNumbers: 11-SRNP-70438; **Taxon:** scientificName: Trigonospila josemariamoragai; phylum: Arthropoda; class: Insecta; order: Diptera; family: Tachinidae; genus: Trigonospila; specificEpithet: josemariamoragai; scientificNameAuthorship: Fleming & Wood, 2015; **Location:** continent: Central America; country: Costa Rica; countryCode: CR; stateProvince: Guanacaste; county: Area de Conservacion Guanacaste; locality: Sector Pitilla; verbatimLocality: Sendero Navarro; **Identification:** identifiedBy: AJ Fleming; dateIdentified: 2015; **Event:** samplingProtocol: reared from caterpillar of Egchiretes Poole01 (Nolidae); verbatimEventDate: 21-Mar-2011; **Record Level:** language: en; institutionCode: CNC; collectionCode: Insects; basisOfRecord: Pinned Specimen
- **Type status:** Paratype. **Occurrence:** occurrenceDetails: http://janzen.sas.upenn.edu; catalogNumber: DHJPAR0042264; recordedBy: D.H. Janzen & W. Hallwachs; individualID: DHJPAR0042264; individualCount: 1; sex: F; lifeStage: adult; preparations: pinned; otherCatalogNumbers: 11-SRNP-70454; **Taxon:** scientificName: Trigonospila josemariamoragai; phylum: Arthropoda; class: Insecta; order: Diptera; family: Tachinidae; genus: Trigonospila; specificEpithet: josemariamoragai; scientificNameAuthorship: Fleming & Wood, 2015; **Location:** continent: Central America; country: Costa Rica; countryCode: CR; stateProvince: Guanacaste; county: Area de Conservacion Guanacaste; locality: Sector Pitilla; verbatimLocality: Sendero Navarro; **Identification:** identifiedBy: AJ Fleming; dateIdentified: 2015; **Event:** samplingProtocol: reared from caterpillar of Egchiretes Poole01 (Nolidae); verbatimEventDate: 20-Mar-2011; **Record Level:** language: en; institutionCode: CNC; collectionCode: Insects; basisOfRecord: Pinned Specimen
- **Type status:** Paratype. **Occurrence:** occurrenceDetails: http://janzen.sas.upenn.edu; catalogNumber: DHJPAR0042265; recordedBy: D.H. Janzen & W. Hallwachs; individualID: DHJPAR0042265; individualCount: 1; sex: F; lifeStage: adult; preparations: pinned; otherCatalogNumbers: 11-SRNP-70426; **Taxon:** scientificName: Trigonospila josemariamoragai; phylum: Arthropoda; class: Insecta; order: Diptera; family: Tachinidae; genus: Trigonospila; specificEpithet: josemariamoragai; scientificNameAuthorship: Fleming & Wood, 2015; **Location:** continent: Central America; country: Costa Rica; countryCode: CR; stateProvince: Guanacaste; county: Area de Conservacion Guanacaste; locality: Sector Pitilla; verbatimLocality: Sendero Navarro; **Identification:** identifiedBy: AJ Fleming; dateIdentified: 2015; **Event:** samplingProtocol: reared from caterpillar of Egchiretes Poole01 (Nolidae); verbatimEventDate: 20-Mar-2011; **Record Level:** language: en; institutionCode: CNC; collectionCode: Insects; basisOfRecord: Pinned Specimen
- **Type status:** Paratype. **Occurrence:** occurrenceDetails: http://janzen.sas.upenn.edu; catalogNumber: DHJPAR0042266; recordedBy: D.H. Janzen & W. Hallwachs; individualID: DHJPAR0042266; individualCount: 1; sex: M; lifeStage: adult; preparations: pinned; otherCatalogNumbers: 11-SRNP-70439; **Taxon:** scientificName: Trigonospila josemariamoragai; phylum: Arthropoda; class: Insecta; order: Diptera; family: Tachinidae; genus: Trigonospila; specificEpithet: josemariamoragai; scientificNameAuthorship: Fleming & Wood, 2015; **Location:** continent: Central America; country: Costa Rica; countryCode: CR; stateProvince: Guanacaste; county: Area de Conservacion Guanacaste; locality: Sector Pitilla; verbatimLocality: Sendero Navarro; **Identification:** identifiedBy: AJ Fleming; dateIdentified: 2015; **Event:** samplingProtocol: reared from caterpillar of Egchiretes Poole01 (Nolidae); verbatimEventDate: 20-Mar-2011; **Record Level:** language: en; institutionCode: CNC; collectionCode: Insects; basisOfRecord: Pinned Specimen
- **Type status:** Paratype. **Occurrence:** occurrenceDetails: http://janzen.sas.upenn.edu; catalogNumber: DHJPAR0042267; recordedBy: D.H. Janzen & W. Hallwachs; individualID: DHJPAR0042267; individualCount: 1; sex: M; lifeStage: adult; preparations: pinned; otherCatalogNumbers: 11-SRNP-70433; **Taxon:** scientificName: Trigonospila josemariamoragai; phylum: Arthropoda; class: Insecta; order: Diptera; family: Tachinidae; genus: Trigonospila; specificEpithet: josemariamoragai; scientificNameAuthorship: Fleming & Wood, 2015; **Location:** continent: Central America; country: Costa Rica; countryCode: CR; stateProvince: Guanacaste; county: Area de Conservacion Guanacaste; locality: Sector Pitilla; verbatimLocality: Sendero Navarro; **Identification:** identifiedBy: AJ Fleming; dateIdentified: 2015; **Event:** samplingProtocol: reared from caterpillar of Egchiretes Poole01 (Nolidae); verbatimEventDate: 19-Mar-2011; **Record Level:** language: en; institutionCode: CNC; collectionCode: Insects; basisOfRecord: Pinned Specimen
- **Type status:** Paratype. **Occurrence:** occurrenceDetails: http://janzen.sas.upenn.edu; catalogNumber: DHJPAR0042268; recordedBy: D.H. Janzen & W. Hallwachs; individualID: DHJPAR0042268; individualCount: 1; sex: M; lifeStage: adult; preparations: pinned; otherCatalogNumbers: 11-SRNP-70434; **Taxon:** scientificName: Trigonospila josemariamoragai; phylum: Arthropoda; class: Insecta; order: Diptera; family: Tachinidae; genus: Trigonospila; specificEpithet: josemariamoragai; scientificNameAuthorship: Fleming & Wood, 2015; **Location:** continent: Central America; country: Costa Rica; countryCode: CR; stateProvince: Guanacaste; county: Area de Conservacion Guanacaste; locality: Sector Pitilla; verbatimLocality: Sendero Navarro; **Identification:** identifiedBy: AJ Fleming; dateIdentified: 2015; **Event:** samplingProtocol: reared from caterpillar of Egchiretes Poole01 (Nolidae); verbatimEventDate: 18-Mar-2011; **Record Level:** language: en; institutionCode: CNC; collectionCode: Insects; basisOfRecord: Pinned Specimen
- **Type status:** Paratype. **Occurrence:** occurrenceDetails: http://janzen.sas.upenn.edu; catalogNumber: DHJPAR0042270; recordedBy: D.H. Janzen & W. Hallwachs; individualID: DHJPAR0042270; individualCount: 1; sex: F; lifeStage: adult; preparations: pinned; otherCatalogNumbers: 11-SRNP-70445; **Taxon:** scientificName: Trigonospila josemariamoragai; phylum: Arthropoda; class: Insecta; order: Diptera; family: Tachinidae; genus: Trigonospila; specificEpithet: josemariamoragai; scientificNameAuthorship: Fleming & Wood, 2015; **Location:** continent: Central America; country: Costa Rica; countryCode: CR; stateProvince: Guanacaste; county: Area de Conservacion Guanacaste; locality: Sector Pitilla; verbatimLocality: Sendero Navarro; **Identification:** identifiedBy: AJ Fleming; dateIdentified: 2015; **Event:** samplingProtocol: reared from caterpillar of Egchiretes Poole01 (Nolidae); verbatimEventDate: 21-Mar-2011; **Record Level:** language: en; institutionCode: CNC; collectionCode: Insects; basisOfRecord: Pinned Specimen
- **Type status:** Paratype. **Occurrence:** occurrenceDetails: http://janzen.sas.upenn.edu; catalogNumber: DHJPAR0042271; recordedBy: D.H. Janzen & W. Hallwachs; individualID: DHJPAR0042271; individualCount: 1; sex: M; lifeStage: adult; preparations: pinned; otherCatalogNumbers: 11-SRNP-70429; **Taxon:** scientificName: Trigonospila josemariamoragai; phylum: Arthropoda; class: Insecta; order: Diptera; family: Tachinidae; genus: Trigonospila; specificEpithet: josemariamoragai; scientificNameAuthorship: Fleming & Wood, 2015; **Location:** continent: Central America; country: Costa Rica; countryCode: CR; stateProvince: Guanacaste; county: Area de Conservacion Guanacaste; locality: Sector Pitilla; verbatimLocality: Sendero Navarro; **Identification:** identifiedBy: AJ Fleming; dateIdentified: 2015; **Event:** samplingProtocol: reared from caterpillar of Egchiretes Poole01 (Nolidae); verbatimEventDate: 21-Mar-2011; **Record Level:** language: en; institutionCode: CNC; collectionCode: Insects; basisOfRecord: Pinned Specimen
- **Type status:** Paratype. **Occurrence:** occurrenceDetails: http://janzen.sas.upenn.edu; catalogNumber: DHJPAR0042273; recordedBy: D.H. Janzen & W. Hallwachs; individualID: DHJPAR0042273; individualCount: 1; sex: F; lifeStage: adult; preparations: pinned; otherCatalogNumbers: 11-SRNP-70453; **Taxon:** scientificName: Trigonospila josemariamoragai; phylum: Arthropoda; class: Insecta; order: Diptera; family: Tachinidae; genus: Trigonospila; specificEpithet: josemariamoragai; scientificNameAuthorship: Fleming & Wood, 2015; **Location:** continent: Central America; country: Costa Rica; countryCode: CR; stateProvince: Guanacaste; county: Area de Conservacion Guanacaste; locality: Sector Pitilla; verbatimLocality: Sendero Navarro; **Identification:** identifiedBy: AJ Fleming; dateIdentified: 2015; **Event:** samplingProtocol: reared from caterpillar of Egchiretes Poole01 (Nolidae); verbatimEventDate: 20-Mar-2011; **Record Level:** language: en; institutionCode: CNC; collectionCode: Insects; basisOfRecord: Pinned Specimen
- **Type status:** Paratype. **Occurrence:** occurrenceDetails: http://janzen.sas.upenn.edu; catalogNumber: DHJPAR0042274; recordedBy: D.H. Janzen & W. Hallwachs; individualID: DHJPAR0042274; individualCount: 1; sex: M; lifeStage: adult; preparations: pinned; otherCatalogNumbers: 11-SRNP-70444; **Taxon:** scientificName: Trigonospila josemariamoragai; phylum: Arthropoda; class: Insecta; order: Diptera; family: Tachinidae; genus: Trigonospila; specificEpithet: josemariamoragai; scientificNameAuthorship: Fleming & Wood, 2015; **Location:** continent: Central America; country: Costa Rica; countryCode: CR; stateProvince: Guanacaste; county: Area de Conservacion Guanacaste; locality: Sector Pitilla; verbatimLocality: Sendero Navarro; **Identification:** identifiedBy: AJ Fleming; dateIdentified: 2015; **Event:** samplingProtocol: reared from caterpillar of Egchiretes Poole01 (Nolidae); verbatimEventDate: 20-Mar-2011; **Record Level:** language: en; institutionCode: CNC; collectionCode: Insects; basisOfRecord: Pinned Specimen
- **Type status:** Paratype. **Occurrence:** occurrenceDetails: http://janzen.sas.upenn.edu; catalogNumber: DHJPAR0042275; recordedBy: D.H. Janzen & W. Hallwachs; individualID: DHJPAR0042275; individualCount: 1; sex: M; lifeStage: adult; preparations: pinned; otherCatalogNumbers: 11-SRNP-70443; **Taxon:** scientificName: Trigonospila josemariamoragai; phylum: Arthropoda; class: Insecta; order: Diptera; family: Tachinidae; genus: Trigonospila; specificEpithet: josemariamoragai; scientificNameAuthorship: Fleming & Wood, 2015; **Location:** continent: Central America; country: Costa Rica; countryCode: CR; stateProvince: Guanacaste; county: Area de Conservacion Guanacaste; locality: Sector Pitilla; verbatimLocality: Sendero Navarro; **Identification:** identifiedBy: AJ Fleming; dateIdentified: 2015; **Event:** samplingProtocol: reared from caterpillar of Egchiretes Poole01 (Nolidae); verbatimEventDate: 21-Mar-2011; **Record Level:** language: en; institutionCode: CNC; collectionCode: Insects; basisOfRecord: Pinned Specimen
- **Type status:** Paratype. **Occurrence:** occurrenceDetails: http://janzen.sas.upenn.edu; catalogNumber: DHJPAR0042278; recordedBy: D.H. Janzen & W. Hallwachs; individualID: DHJPAR0042278; individualCount: 1; sex: F; lifeStage: adult; preparations: pinned; otherCatalogNumbers: 11-SRNP-70427; **Taxon:** scientificName: Trigonospila josemariamoragai; phylum: Arthropoda; class: Insecta; order: Diptera; family: Tachinidae; genus: Trigonospila; specificEpithet: josemariamoragai; scientificNameAuthorship: Fleming & Wood, 2015; **Location:** continent: Central America; country: Costa Rica; countryCode: CR; stateProvince: Guanacaste; county: Area de Conservacion Guanacaste; locality: Sector Pitilla; verbatimLocality: Sendero Navarro; **Identification:** identifiedBy: AJ Fleming; dateIdentified: 2015; **Event:** samplingProtocol: reared from caterpillar of Egchiretes Poole01 (Nolidae); verbatimEventDate: 18-Mar-2011; **Record Level:** language: en; institutionCode: CNC; collectionCode: Insects; basisOfRecord: Pinned Specimen
- **Type status:** Paratype. **Occurrence:** occurrenceDetails: http://janzen.sas.upenn.edu; catalogNumber: DHJPAR0052422; recordedBy: D.H. Janzen & W. Hallwachs; individualID: DHJPAR0052422; individualCount: 1; sex: F; lifeStage: adult; preparations: pinned; otherCatalogNumbers: 13-SRNP-76881; **Taxon:** scientificName: Trigonospila josemariamoragai; phylum: Arthropoda; class: Insecta; order: Diptera; family: Tachinidae; genus: Trigonospila; specificEpithet: josemariamoragai; scientificNameAuthorship: Fleming & Wood, 2015; **Location:** continent: Central America; country: Costa Rica; countryCode: CR; stateProvince: Guanacaste; county: Area de Conservacion Guanacaste; locality: Sector Rincon Rain Forest; verbatimLocality: Quebrada Bambu; verbatimElevation: 109; verbatimLatitude: 10.9301; verbatimLongitude: -85.25205; verbatimCoordinateSystem: Decimal; decimalLatitude: 10.9301; decimalLongitude: -85.25205; **Identification:** identifiedBy: AJ Fleming; dateIdentified: 2015; **Event:** samplingProtocol: reared from caterpillar of Egchiretes Poole01 (Nolidae); verbatimEventDate: 08-Aug-2013; **Record Level:** language: en; institutionCode: CNC; collectionCode: Insects; basisOfRecord: Pinned Specimen

#### Description

Male (Fig. 3a, b, c), 8.8 mm. Head (Fig. 3b): frontal vitta dark black, narrowed apically to equal width of the ocellar triangle, parafrontal (as measured between the inner margin of the eye and the frons, at the apex of the lunule) subequal in with to the frontal vitta; frontal bristles arise no lower than level of first antennal segment; antennae black; frontoorbital plate entirely gold; parafacial silvery to slightly gold tinged; palpi black gray; gena 1/8 height of head. Thorax (Fig. 3a): yellow when viewed dorsally with four longitudinal black vittae, these becoming fused postsuturally, appearing as two indistinct blotches covering 2/3^rds^ of thorax post-suturally; three postsutural dorsocentral bristles; scutellum bearing white or yellowish pruinosity over its entirety (occupying 1/2 or more of total area); 3 pairs of scutellar marginal bristles; subapical scutellars widely divergent, lateral scutellars hairlike, closer to apex, than to basal scutellars; legs black. Wings: pale smoky grayish in color, with one bristle arising at the joint between R_1_ and R_2+3_. Abdomen (Figs 1c, 3a): ST1+2, dark velvety black with very slight infiltration of yellow band from next tergite, along its posterior margin, T3, T4, and T5, all with bright, yellow bands covering 1/3^rd^ or more of tergal surface arising at the margins of between the abdominal tergites, these bands wrapping around to the underside; bright yellow bands straddling the margin between tergites T1+2, T3, and the anterior margin of T4; tergal bands possessing a sharp mid-dorsal peak figuring prominently on both T3 and T4, these extending 1/2 way across T3, and to the margin of T4; T3 and T4 possessing 2 pairs of medial discal bristles, insertion point of abdominal bristles punctuated by a black outline appearing as black spots.

Female (Fig. 3d, e, f), 6.7 mm. Head: frontal vitta dark black, parallel sided apically equal to twice the width of the ocellar triangle, parafrontal equal in width to the frontal vitta; frontal bristles arise no lower than level of first antennal segment; proclinate orbital bristles present; antennae black; frontoorbital plate almost entirely gold; parafacial silvery to slightly gold tinged; palpi orange, slightly haired along upper surface; gena 1/10 height of head. Thorax (Fig. 3d): yellow when viewed dorsally with four longitudinal black vittae, these becoming fused postsuturally, appearing as two indistinct blotches covering just over 1/2 of thorax postsuturally; three postsutural dorsocentral bristles; scutellum bearing white or yellowish pruinosity over its entirety (occupying 1/2 or more of total area); 3 pairs of scutellar marginal bristles; subapical scutellars widely divergent, lateral scutellars still reduced but more pronounced than in males, almost half the length of the subapicals, these closer to apex, than to basal scutellars; legs black with silvery sheen. Wings: pale smoky grayish in color, with one bristle arising at the joint between R_1_ and R_2+3_. Abdomen (Figs 2d, 3f): pointed downward apically so as to appear strongly curved; ST1+2, dark velvety black with very slight infiltration of dull, grayish or bright yellow bands from next segment along posterior margin, T3, T4, and T5 with bands covering up to 1/2 of tergal surface; bands peaked dorsocentrally creating an apparent dorsocentral stripe extending the length of the abdomen (Fig. 2d); abdominal bands wrapping around to the underside; bright yellow bands straddling the margin between tergites ST1+2, T3, with yellow extending up to but not above median marginal bristles on ST1+2 and the anterior margin of T4; T3 and T4 possessing 1 pair of medial discal bristles, insertion point of abdominal bristles punctuated by a black outline appearing as black spots; when viewed dorsally abdomen appearing to have 4 black rounded triangles surrounded by yellow, with 4 black dots between them.

#### Diagnosis

Small black and yellow fly, with 4 prominent black stripes on the thorax, these smudging together so that it appears as 2 large thoracic vittae and a golden scutellum. Males with a straight conical, and apically pointed abdomen, with 3 gold bands interspersed with black wrapping the abdomen, terminating in a black tip. Female abdomen with a strong down-pointing curve abdominal bands mid-dorsally pointed joining the next segment’s gold band so that 4 small black triangles become apparent on abdomen.

#### Etymology

*Trigonospila josemariamoragai* is named in honor of Jose Mario Moraga, in recognition of his frequent rescues of ACG parataxonomists' computers.

#### Distribution

Costa Rica, ACG, Prov. Guanacaste, rain forest.

#### Ecology

Reared from, Nolidae, *Steniscadia polyodonta*; Elachistidae, *Stenoma* spp. (19 records). One fly larva per caterpillar.

### Trigonospila uniformis

Fleming & Wood sp. n.

urn:lsid:zoobank.org:act:C1DF4394-DD67-4553-9E5B-0EFD3F7D6AE2

#### Materials

- **Type status:** Holotype. **Occurrence:** occurrenceDetails: http://janzen.sas.upenn.edu; catalogNumber: DHJPAR0035709; recordedBy: D.H. Janzen & W. Hallwachs; individualID: DHJPAR0035709; individualCount: 1; sex: M; lifeStage: adult; preparations: pinned; otherCatalogNumbers: 09-SRNP-44688; **Taxon:** scientificName: Trigonospila uniformis; phylum: Arthropoda; class: Insecta; order: Diptera; family: Tachinidae; genus: Trigonospila; specificEpithet: uniformis; scientificNameAuthorship: Fleming & Wood, 2015; **Location:** continent: Central America; country: Costa Rica; countryCode: CR; stateProvince: Alajuela; county: Area de Conservacion Guanacaste; locality: Sector Rincon Rain Forest; verbatimLocality: Estacion Llanura; verbatimElevation: 135; verbatimLatitude: 10.933; verbatimLongitude: -85.253; verbatimCoordinateSystem: Decimal; decimalLatitude: 10.933; decimalLongitude: -85.253; **Identification:** identifiedBy: AJ Fleming; dateIdentified: 2015; **Event:** samplingProtocol: reared from caterpillar of Stenoma Janzen44 (Elachistidae); verbatimEventDate: Jul-15-2009; **Record Level:** language: en; institutionCode: CNC; collectionCode: Insects; basisOfRecord: Pinned Specimen
- **Type status:** Paratype. **Occurrence:** occurrenceDetails: http://janzen.sas.upenn.edu; catalogNumber: DHJPAR0040672; recordedBy: D.H. Janzen & W. Hallwachs; individualID: DHJPAR0040672; individualCount: 1; sex: F; lifeStage: adult; preparations: pinned; otherCatalogNumbers: 10-SRNP-75629; **Taxon:** scientificName: Trigonospila uniformis; phylum: Arthropoda; class: Insecta; order: Diptera; family: Tachinidae; genus: Trigonospila; specificEpithet: uniformis; scientificNameAuthorship: Fleming & Wood, 2015; **Location:** continent: Central America; country: Costa Rica; stateProvince: Alajuela; county: Area de Conservacion Guanacaste; locality: Sector Rincon Rain Forest; verbatimLocality: Quebrada Bambu; verbatimElevation: 109; verbatimLatitude: 10.93; verbatimLongitude: -85.252; verbatimCoordinateSystem: Decimal; decimalLatitude: 10.93; decimalLongitude: -85.252; **Identification:** identifiedBy: AJ Fleming; dateIdentified: 2015; **Event:** samplingProtocol: reared from caterpillar of Antaeotricha spurca (Elachistidae); verbatimEventDate: 26-Apr-2010; **Record Level:** language: en; institutionCode: CNC; collectionCode: Insects; basisOfRecord: Pinned Specimen

#### Description

Male (Fig. 4a, b, c), 9 mm. Head (Fig. 4b): frontal vitta dark black, slightly tapered apically to twice the width of the ocellar triangle, parafrontal 1/2 as wide as frontal vitta; frontal bristles arise no lower than level of first antennal segment; antennae black; frontoorbital plate silvery-gold turning to black apically; parafacial silvery to slightly gold tinged; palpi black gray; gena 1/8 height of head. Thorax (Fig. 4a): yellow when viewed dorsally with four longitudinal black vittae, these appear fused throughout their length with only slight separation apparent; appearing as two indistinct blotches covering 2/3^rds^ of thorax postsuturally; three postsutural dorsocentral bristles; scutellum bearing white or yellowish pruinosity only at apex (occupying less than 1/5^th^ of total area); 3 pairs of scutellar marginal bristles; subapical scutellars widely divergent, lateral scutellars reduced, almost half the length of the subapicals, these closer to apex, than to basal scutellars; legs black. Wings: pale smoky grayish in color, with one bristle arising at the joint between R_1_ and R_2+3_. Abdomen (Figs 1a, 4a): abdominal tergites dark velvety black, with bright, yellow bands covering less than 1/3^rd^ of tergal surface arising at the margins of between the abdominal tergites, these bands not wrapping around to the underside; bright yellow bands straddling the margin between tergites ST1+2, T3, and the anterior margin of T4; tergal bands not possessing a sharp mid-dorsal peak rather appearing flat.

Female (Fig. 4d, e, f), 4 mm. Head (Fig. 4e): frontal vitta dark tawny, parallel sided apically equal to twice the width of the ocellar triangle, parafrontal 1.5 times as wide as frontal vitta; frontal bristles arise no lower than level of first antennal segment; proclinate orbital bristles present; antennae light black with orange present at base of first flagellomere; frontoorbital plate entirely gold; parafacial narrow, silvery to slightly gold tinged; palpi light gray at base, with orange tips, slightly haired along upper surface; gena 1/10 height of head. Thorax (Fig. 3d): yellow when viewed dorsally with four longitudinal black vittae, these becoming remaining separate postsuturally, appearing as four distinct lines covering just over 1/2 of thorax postsuturally; three postsutural dorsocentral bristles; scutellum bearing white or yellowish pruinosity over half of its area; scutellar bristles similar to males. Wings: pale smoky grayish in color, with one bristle arising at the joint between R_1_ and R_2+3_. Abdomen (Figs 2a, 4d): pointed downward apically so as to appear strongly curved; abdominal tergites dark velvety black, with dull, grayish bands covering at least 1/2 of tergal surface; bands flat and with no distinctive mid-dorsal peaks (Fig. 2a); abdominal bands wrapping around to the underside; bright yellow bands straddling the margin between tergites ST1+2, and T3 with yellow-gray color extending up to and beyond insertion point of median marginal bristles on ST1+2; T3 and T4 possessing 1 pair of medial discal bristles, insertion point of abdominal bristles punctuated by a black outline appearing as black spots.

#### Diagnosis

Small black and yellow fly, with 4 prominent black stripes on the thorax, these smudging together so that it appears as 2 large thoracic vittae. Males have a black scutellum, straight conical, and apically pointed abdomen, with 3 narrow gold bands interspersed with black wrapping the abdomen, terminating in a black tip. Female abdomen with a strong down-pointing curve abdominal, 3 grayish abdominal bands lacking mid-dorsal point.

#### Etymology

From the Latin “*uniformis*”, for not changing in form or character, in reference to the uniform nature of the pruinose bands on the abdomen.

#### Distribution

Costa Rica, ACG, Prov. Alajuela, rain forest, 109–135 m elevation.

#### Ecology

Reared from, Elachistidae, *Stenoma* Janzen44 and *Antaeotricha spurca* (2 records). One fly larva per caterpillar.

### Trigonospila edwinbermudezi

Fleming & Wood sp. n.

urn:lsid:zoobank.org:act:231FE687-64FF-432B-9A02-A38DE8F9C542

#### Materials

- **Type status:** Holotype. **Occurrence:** occurrenceDetails: http://janzen.sas.upenn.edu; catalogNumber: DHJPAR0056126; recordedBy: D.H. Janzen & W. Hallwachs; individualID: DHJPAR0056126; individualCount: 1; sex: M; lifeStage: adult; preparations: pinned; otherCatalogNumbers: 14-SRNP-71290; **Taxon:** scientificName: Trigonospila edwinbermudezi; phylum: Arthropoda; class: Insecta; order: Diptera; family: Tachinidae; genus: Trigonospila; specificEpithet: edwinbermudezi; scientificNameAuthorship: Fleming & Wood, 2015; **Location:** continent: Central America; country: Costa Rica; countryCode: CR; stateProvince: Guanacaste; county: Area de Conservacion Guanacaste; locality: Sector Pitilla; verbatimLocality: Medrano; verbatimElevation: 380; verbatimLatitude: 11.01602; verbatimLongitude: -85.38053; verbatimCoordinateSystem: Decimal; decimalLatitude: 11.01602; decimalLongitude: -85.38053; **Identification:** identifiedBy: AJ Fleming; dateIdentified: 2015; **Event:** samplingProtocol: reared from caterpillar of Paridnea holophaealis (Pyralidae); verbatimEventDate: Aug-16-2014; **Record Level:** language: en; institutionCode: CNC; collectionCode: Insects; basisOfRecord: Pinned Specimen
- **Type status:** Paratype. **Occurrence:** occurrenceDetails: http://janzen.sas.upenn.edu; catalogNumber: DHJPAR0018447; recordedBy: D.H. Janzen & W. Hallwachs; individualID: DHJPAR0018447; individualCount: 1; sex: F; lifeStage: adult; preparations: pinned; otherCatalogNumbers: 02-SRNP-28022; **Taxon:** scientificName: Trigonospila edwinbermudezi; phylum: Arthropoda; class: Insecta; order: Diptera; family: Tachinidae; genus: Trigonospila; specificEpithet: edwinbermudezi; scientificNameAuthorship: Fleming & Wood, 2015; **Location:** continent: Central America; country: Costa Rica; countryCode: CR; stateProvince: Guanacaste; county: Area de Conservacion Guanacaste; locality: Sector El Hacha; verbatimLocality: Finca Araya; verbatimElevation: 295; verbatimLatitude: 11.015; verbatimLongitude: -85.511; verbatimCoordinateSystem: Decimal; decimalLatitude: 11.015; decimalLongitude: -85.511; **Identification:** identifiedBy: AJ Fleming; dateIdentified: 2015; **Event:** samplingProtocol: reared from caterpillar of Omphalocera cariosa (Pyralidae); verbatimEventDate: Sep-11-2002; **Record Level:** language: en; institutionCode: CNC; collectionCode: Insects; basisOfRecord: Pinned Specimen
- **Type status:** Paratype. **Occurrence:** occurrenceDetails: http://janzen.sas.upenn.edu; catalogNumber: DHJPAR0044883; recordedBy: D.H. Janzen & W. Hallwachs; individualID: DHJPAR0044883; individualCount: 1; sex: F; lifeStage: adult; preparations: pinned; otherCatalogNumbers: 11-SRNP-2384; **Taxon:** scientificName: Trigonospila edwinbermudezi; phylum: Arthropoda; class: Insecta; order: Diptera; family: Tachinidae; genus: Trigonospila; specificEpithet: edwinbermudezi; scientificNameAuthorship: Fleming & Wood, 2015; **Location:** continent: Central America; country: Costa Rica; countryCode: CR; stateProvince: Guanacaste; county: Area de Conservacion Guanacaste; locality: Sector San Cristobal; verbatimLocality: Quebrada Garcia; verbatimElevation: 495; verbatimLatitude: 10.861; verbatimLongitude: -85.426; verbatimCoordinateSystem: Decimal; decimalLatitude: 10.861; decimalLongitude: -85.426; **Identification:** identifiedBy: AJ Fleming; dateIdentified: 2015; **Event:** samplingProtocol: reared from caterpillar of Paridnea holophaealis (Pyralidae); verbatimEventDate: 03-Aug-2011; **Record Level:** language: en; institutionCode: CNC; collectionCode: Insects; basisOfRecord: Pinned Specimen

#### Description

Male (Fig. 5a, b, c), 9 mm. Head (Fig. 5b): frontal vitta dark black, tapered apically approximately 1.5X the width of the ocellar triangle, parafrontal 1/2 as wide as frontal vitta; frontal bristles arise no lower than level of pedicel; antennae black; frontoorbital plate gold turning to black apically; parafacial gold tinged in its entirety; palpi black gray; facial ridge at level of vibrissa with distinct black tinge; gena 1/6 height of head. Thorax (Fig. 5a): yellow-gold when viewed dorsally with four longitudinal black vittae, appearing fused throughout their length; appearing as two indistinct blotches covering 2/3^rds^ of thorax postsuturally; three postsutural dorsocentral bristles; scutellum bearing yellowish pruinosity over 1/2 its total area; 3 pairs of scutellar marginal bristles; subapical scutellars widely divergent, lateral scutellars half the length of suabpicals, with these arising almost adjacent to subapical scutellars; legs black. Wings: dark smoky grayish in color, with one bristle arising at the joint between R_1_ and R_2+3; _well developped costal spine. Abdomen (Figs 1d, 5a, c): ST1+2 dark velvety black, with slight yellow extending from anterior edge of following tergite, T3, T4, and T5, with bright, yellow bands covering approximately 1/3^rd^ of tergal surface arising at the margins of between the abdominal tergites, these bands wrapping around sides of abdomen but not the underside; bright yellow bands straddling the margin between tergites ST1+2, T3, T4 and over 50% of T5; tergal bands possessing a sharp mid-dorsal peak extending almost to margin of adjacent tergite, in both T3 and T4, but not reaching margin.

Female (Fig. 5d, e, f), 10 mm. Head (Fig. 5e): frontal vitta dark black, parallel sided apically equal to twice the width of the ocellar triangle, prarafrontal 1.5 times as wide as frontal vitta; frontal bristles arise no lower than level of first antennal segment; proclinate orbital bristles present; antennae black; frontoorbital plate entirely gold; parafacial silvery to slightly gold tinged; palpi orange, slightly haired along upper surface forming a hirsute oval along the upper surface of the palps; eyes bearing very short sparse hairs; gena 1/5 height of head. Thorax (Fig. 5d): yellow when viewed dorsally with four longitudinal black vittae, these becoming fused postsuturally, appearing as two indistinct blotches covering just over 1/2 of thorax postsuturally; three postsutural dorsocentral bristles; scutellum bearing yellowish pruinosity over its entirety (occupying 1/2 or more of total area); 3 pairs of scutellar marginal bristles; subapical scutellars widely divergent, lateral scutellars, almost 2/3^rds^ the length of the subapicals, these closer to apex, than to basal scutellars; legs black with silvery sheen. Wings: pale smoky grayish in color, with one bristle arising at the joint between R_1_ and R_2+3_. Abdomen (Figs 2c, 5d, f): pointed downward apically so as to appear strongly curved; ST1+2, dark velvety black with very slight infiltration of dull, grayish or bright yellow bands from next segment along posterior margin, T3, T4, and T5 with dull, grayish or bright yellow bands covering at up to 1/2 of tergal surface; bands peaked dorsocentrally creating an apparent dorsocentral stripe extending the length of the abdomen; abdominal bands wrapping around to the underside; bright yellow bands straddling the margin between tergites ST1+2 and T3 with yellow extending up to but not above median marginal bristles on ST1+2, and the anterior margin of T4; T3 and T4 possessing 1 pair of medial discal bristles, insertion point of abdominal bristles punctuated by a black outline appearing as black spots; when viewed dorsally abdomen appearing to have 4 black triangles surrounded by yellow, with 4 black dots between them.

#### Diagnosis

Small black and gold fly, with 4 prominent black stripes on the thorax, appearing as 2 large thoracic vittae with a golden divider, and a golden scutellum. Female abdomen with a strong down-pointing curve abdominal bands mid-dorsally pointed joining the next segment’s gold band so that 4 small black triangles become apparent on abdomen. The tip of the abomen golden.

#### Etymology

*Trigonospila edwinbermudezi* is named in honor of Edwin Bermudez, the first Encargado de Sector for Sector El Hacha of ACG.

#### Distribution

Costa Rica, ACG, Prov. Guanacaste, rain forest and dry forest-rain forest interface, 295–495 m elevation.

#### Ecology

Reared from, Pyralidae, *Omphalocera cariosa* and *Paridnea holophaealis* (3 records). One fly larva per caterpillar.

### Trigonospila panamensis

(Townsend, 1919)

#### Materials

- **Type status:** Other material. **Occurrence:** occurrenceDetails: http://janzen.sas.upenn.edu; catalogNumber: DHJPAR0018446; recordedBy: D.H. Janzen & W. Hallwachs; individualID: DHJPAR0018446; individualCount: 1; sex: F; lifeStage: adult; preparations: pinned; otherCatalogNumbers: 96-SRNP-9740; **Taxon:** scientificName: Trigonospila panamensis; phylum: Arthropoda; class: Insecta; order: Diptera; family: Tachinidae; genus: Trigonospila; specificEpithet: panamensis; scientificNameAuthorship: Townsend, 1919; **Location:** continent: Central America; country: Costa Rica; countryCode: CR; stateProvince: Guanacaste; county: Sector Horizontes; locality: Area de Conservacion Guanacaste; verbatimLocality: Sitio La Dama; verbatimElevation: 105; verbatimLatitude: 10.786; verbatimLongitude: -85.558; verbatimCoordinateSystem: Decimal; decimalLatitude: 10.786; decimalLongitude: -85.558; **Identification:** identifiedBy: AJ Fleming; dateIdentified: 2015; **Event:** samplingProtocol: Host Collection; verbatimEventDate: 04-Oct-1996; **Record Level:** language: en; institutionCode: CNC; collectionCode: Insects; basisOfRecord: Pinned Specimen
- **Type status:** Other material. **Occurrence:** occurrenceDetails: http://janzen.sas.upenn.edu; catalogNumber: DHJPAR0035626; recordedBy: D.H. Janzen & W. Hallwachs; individualID: DHJPAR0035626; individualCount: 1; sex: F; lifeStage: adult; preparations: pinned; otherCatalogNumbers: 09-SRNP-13144; **Taxon:** scientificName: Trigonospila panamensis; phylum: Arthropoda; class: Insecta; order: Diptera; family: Tachinidae; genus: Trigonospila; specificEpithet: panamensis; scientificNameAuthorship: Townsend, 1919; **Location:** continent: Central America; country: Costa Rica; stateProvince: Guanacaste; county: Sector Santa Rosa; locality: Area de Conservacion Guanacaste; verbatimLocality: Cafetal; verbatimElevation: 280; verbatimLatitude: 10.858; verbatimLongitude: -85.611; verbatimCoordinateSystem: Decimal; decimalLatitude: 10.858; decimalLongitude: -85.611; **Identification:** identifiedBy: AJ Fleming; dateIdentified: 2015; **Event:** samplingProtocol: Host Collection; verbatimEventDate: 26-Jun-2009; **Record Level:** language: en; institutionCode: CNC; collectionCode: Insects; basisOfRecord: Pinned Specimen
- **Type status:** Other material. **Occurrence:** occurrenceDetails: http://janzen.sas.upenn.edu; catalogNumber: DHJPAR0036435; recordedBy: D.H. Janzen & W. Hallwachs; individualID: DHJPAR0036435; individualCount: 1; sex: M; lifeStage: adult; preparations: pinned; otherCatalogNumbers: 09-SRNP-13124; **Taxon:** scientificName: Trigonospila panamensis; phylum: Arthropoda; class: Insecta; order: Diptera; family: Tachinidae; genus: Trigonospila; specificEpithet: panamensis; scientificNameAuthorship: Townsend, 1919; **Location:** continent: Central America; country: Costa Rica; stateProvince: Guanacaste; county: Sector Santa Rosa; locality: Area de Conservacion Guanacaste; verbatimLocality: Cafetal; verbatimElevation: 280; verbatimLatitude: 10.858; verbatimLongitude: -85.611; verbatimCoordinateSystem: Decimal; decimalLatitude: 10.858; decimalLongitude: -85.611; **Identification:** identifiedBy: AJ Fleming; dateIdentified: 2015; **Event:** samplingProtocol: Host Collection; verbatimEventDate: 16-Jun-2009; **Record Level:** language: en; institutionCode: CNC; collectionCode: Insects; basisOfRecord: Pinned Specimen
- **Type status:** Other material. **Occurrence:** occurrenceDetails: http://janzen.sas.upenn.edu; catalogNumber: DHJPAR0036444; recordedBy: D.H. Janzen & W. Hallwachs; individualID: DHJPAR0036444; individualCount: 1; sex: M; lifeStage: adult; preparations: pinned; otherCatalogNumbers: 09-SRNP-13112; **Taxon:** scientificName: Trigonospila panamensis; phylum: Arthropoda; class: Insecta; order: Diptera; family: Tachinidae; genus: Trigonospila; specificEpithet: panamensis; scientificNameAuthorship: Townsend, 1919; **Location:** continent: Central America; country: Costa Rica; stateProvince: Guanacaste; county: Sector Santa Rosa; locality: Area de Conservacion Guanacaste; verbatimLocality: Cafetal; verbatimElevation: 280; verbatimLatitude: 10.858; verbatimLongitude: -85.611; verbatimCoordinateSystem: Decimal; decimalLatitude: 10.858; decimalLongitude: -85.611; **Identification:** identifiedBy: AJ Fleming; dateIdentified: 2015; **Event:** samplingProtocol: Host Collection; verbatimEventDate: 15-Jun-2009; **Record Level:** language: en; institutionCode: CNC; collectionCode: Insects; basisOfRecord: Pinned Specimen
- **Type status:** Other material. **Occurrence:** occurrenceDetails: http://janzen.sas.upenn.edu; catalogNumber: DHJPAR0042319; recordedBy: D.H. Janzen & W. Hallwachs; individualID: DHJPAR0042319; individualCount: 1; sex: M; lifeStage: adult; preparations: pinned; otherCatalogNumbers: 11-SRNP-67043; **Taxon:** scientificName: Trigonospila panamensis; phylum: Arthropoda; class: Insecta; order: Diptera; family: Tachinidae; genus: Trigonospila; specificEpithet: panamensis; scientificNameAuthorship: Townsend, 1919; **Location:** continent: Central America; country: Costa Rica; stateProvince: Alajuela; county: Sector Rincon Rain Forest; locality: Area de Conservacion Guanacaste; verbatimLocality: Baches; **Identification:** identifiedBy: AJ Fleming; dateIdentified: 2015; **Event:** samplingProtocol: Host Collection; verbatimEventDate: 15-Feb-2011; **Record Level:** language: en; institutionCode: CNC; collectionCode: Insects; basisOfRecord: Pinned Specimen

#### Description

Male, previously unknown from the original description of *T. panamensis* Townsend 1919 (Fig. 6a, b, c), 8 mm. Head (Fig. 6b): frontal vitta dark black, narrowly tapered apically to just slightly greater than the width of the ocellar triangle, parafrontal as wide as frontal vitta; frontal bristles arise no lower than level of first antennal segment; antennae black; frontoorbital plate silvery-gold turning to black apically; parafacial silvery to slightly gold tinged; palpi black gray with orange tips; gena 1/10 height of head. Thorax (Fig. 6a): yellow when viewed dorsally with four longitudinal black vittae, these appear to be distinct and separate throughout their length; dorsal lines remain separate post suturally covering 2/3^rds^ of thorax; three postsutural dorsocentral bristles; scutellum bearing white or yellowish pruinosity occupying ½ or more of total area; 3 pairs of scutellar marginal bristles; subapical scutellars widely divergent, lateral scutellars reduced, almost half the length of the subapicals, these closer to apex, than to basal scutellars; legs black. Wings: pale smoky grayish in color, with one bristle arising at the joint between R_1_ and R_2+3_. Abdomen (Figs 1b, 6a): ST1+2, dark velvety black with very slight infiltration of yellow band from next tergite, along its posterior margin, T3, T4, and T5, all with bright, narrow yellow bands covering 1/3^rd^ or more of tergal surface, these bands wrapping around to underside of tergites; bright yellow bands straddling the margin between tergites ST1+2, T3, and the anterior margin of T4; tergal bands not possessing a sharp mid-dorsal peak, instead the margins of the bands appear as jagged on both T3 and T4; yellow bands wrapping around to the underside of the abdomen.

Female (Fig. 6d, e, f), 5.5 mm. Head (Fig. 6e): frontal vitta dark tawny, parallel sided apically equal to twice the width of the ocellar triangle, parafrontal 1.5 times as wide as frontal vitta; frontal bristles arise no lower than level of first antennal segment; proclinate orbital bristles present; antennae with orange present throughout first flagellomere; frontoorbital plate gold up to last proclinate orbital then turning to silver; parafacial narrow, silvery tinged; palpi orange tips, slightly haired along upper surface; gena 1/10 height of head. Thorax (Fig. 6d): yellow when viewed dorsally with four longitudinal black vittae, outer lines appear shorter than inner pair pre-suturally, these remaining separate postsuturally, appearing as four distinct lines covering just over 1/2 of thorax postsuturally; three postsutural dorsocentral bristles; scutellum bearing white or yellowish pruinosity over all of its area; 3 pairs of scutellar marginal bristles; subapical scutellars widely divergent, lateral scutellars reduced, almost half the length of the subapicals, these closer to apex, than to basal scutellars. Wings: pale smoky grayish in color, with one bristle arising at the joint between R_1_ and R_2+3_. Abdomen (Figs 2b, 6d): pointed downward apically so as to appear strongly curved; ST1+2, dark velvety black with very slight infiltration of dull, grayish or bright yellow bands from next segment along posterior margin, T3, T4, and T5 with dull, grayish bands covering extending to cover more than ½ of T3, banding on T4 covering all but 1/5^th^ of tergal surface; abdominal bands wrapping around to the underside which is entirely gray; bright yellow bands straddling the margin between ST1+2 and T3 with yellow-gray color extending up to and beyond insertion point of median marginal bristles on ST1+2; T3 and T4 possessing 1 pair of medial discal bristles, insertion point of abdominal bristles punctuated by a black outline appearing as black spots.

The authors wish to caution that this species-level identification is based solely on morphology, since no DNA barcoded specimens of *T. panamensis* are available for molecular comparison.

#### Diagnosis

Small black and gray fly, with 4 prominent black stripes on the thorax, these do not smudge together and remain distinctively separate in females, scutellum gold. Males with a straight conical, and apically pointed abdomen, with 3 gold bands interspersed with black wrapping the abdomen, terminating in a black tip. Female abdomen with a strong down-pointing curved abdomen, abdominal bands mid-dorsally pointed joining the next segment’s gold band so that 4 small black dashes become apparent on abdomen.

#### Distribution

Panama, Taboga Island; Costa Rica, ACG, Prov. Alajuela and Guanacaste, rain forest and dry forest, 105–280 m elevation.

#### Ecology

Reared from, Crambidae, Elachistidae, Tortricidae, and Pyralidae (7 records). One fly larva per caterpillar.

## Identification Keys

### Key to the species of *Trigonospila* reared from caterpillars in Area de Conservación Guanacaste, Northwestern Costa Rica

**Table:** 

1	Proclinate orbital bristles present (♀) (Figs 3e, 4e, 5b, 6e).	2
–	Proclinate orbital bristles absent (♂) (Figs 3b, 4b, 6b).	5
2	Abdominal tergites dark velvety black, with dull, grayish bands covering up to 1/2 of tergal surface; bands are transverse with no distinctive mid-dorsal extension posteriorly (Fig. 2a	*T. uniformis* **sp. n.**
–	Abdominal tergites dark velvety black, with dull, grayish or bright yellow bands covering at least 1/2 of tergal surface; transverse bands possessing mid-dorsal extensions posteriorly creating an apparent dorsocentral stripe extending the length of the abdomen (Fig. 2b, c, d).	3
3	Abdominal banding extending to cover more than ½ of T3, banding on T4 covering all but 1/5^th^ of tergal surface (Fig. 2b).	*T. panamensis* **(Townsend)**
–	Abdominal banding extending to cover up to ½ of T3, and T4, when coupled with dorsocentral stripe 4 black triangles become evident (Fig. 2c, d).	4
4	Abdominal banding extending out onto posterior margin of ST1+2 extending beyond the insertion point of median marginal bristles on ST1+2 (Fig. 2c)	*T. edwinbermudezi* **sp. n.**
–	Abdominal banding extending out onto posterior margin of ST1+2 extending up to but not beyond the insertion point of median marginal bristles on ST1+2 (Fig. 2d)	*T. josemariamoragai* **sp. n.**
5	Scutellum with white pruinosity only at tip (occupying 1/3 or less of total area); abdominal tergites dark velvety black, with bright, narrow yellow bands covering up to 1/5^th^ of tergal surface, bands not wrapping around to underside of tergites; bright yellow bands straddling the margin between ST1+2 and T3, and the anterior margin of T4 flat, with no distinctive mid-dorsal peaks (Fig. 1a).	*T. uniformis* **sp. n.**
–	Scutellum bearing white or yellowish pruinosity over 2/3 or more of total area; abdominal tergites dark velvety black, with bright, narrow yellow bands covering 1/3^rd^ or more of tergal surface, bands wrapping around to underside of tergites; bright yellow bands straddling the margin between ST1+2 and T3, and the anterior margin of T4, either with rough edging or the presence of a distinct mid-dorsal peak (Fig. 1b, c).	6
6	Tergal bands not possessing a sharp mid-dorsal peak, instead the margins of the bands appearing as jagged on both T3 and T4.	*T. panamensis* **(Townsend)**
–	Tergal bands possessing a sharp mid-dorsal peak figuring prominently on both T3 and T4.	7
7	Mid-dorsal peak extending almost to hind margin of T3; parafacial with no traces of silver; thoracic vittae fused throughout their entire length.	*T. josemariamoragai* **sp. n.**
–	Mid-dorsal peak not extending to hind margin T3 of adjacent tergite; parafacial with silver on lower half; thoracic vittae not fused pre-suturally.	*T. edwinbermudezi* **sp. n.**

This key was prepared based on the specimens collected as a result of the 40+ year inventory still being conducted in ACG. Our key is intended to identify the fauna present within the confines of the ACG.

## Supplementary Material

Supplementary material 1 NJ tree of ACG inventory Trigonospila Data type: phylogenetic tree
Brief description: Neighbor-joining tree of DNA barcodes from ACG inventory *Trigonospila* as of January, 2015. The inventory is ongoing and as new specimens are added, they can be accessed on BOLD.
File: oo_36912.pdf Fleming et al.

XML Treatment for Trigonospila josemariamoragai

XML Treatment for Trigonospila uniformis

XML Treatment for Trigonospila edwinbermudezi

XML Treatment for Trigonospila panamensis

## Figures and Tables

**Figure 1a.**
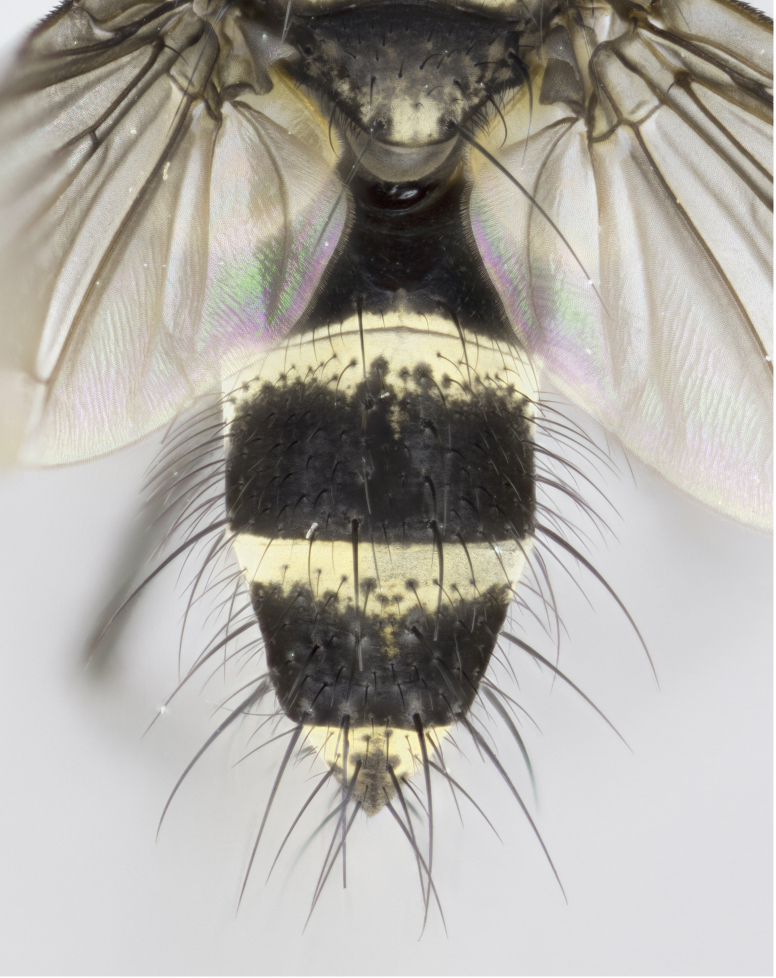
*T. uniformis* **sp. n.**

**Figure 1b.**
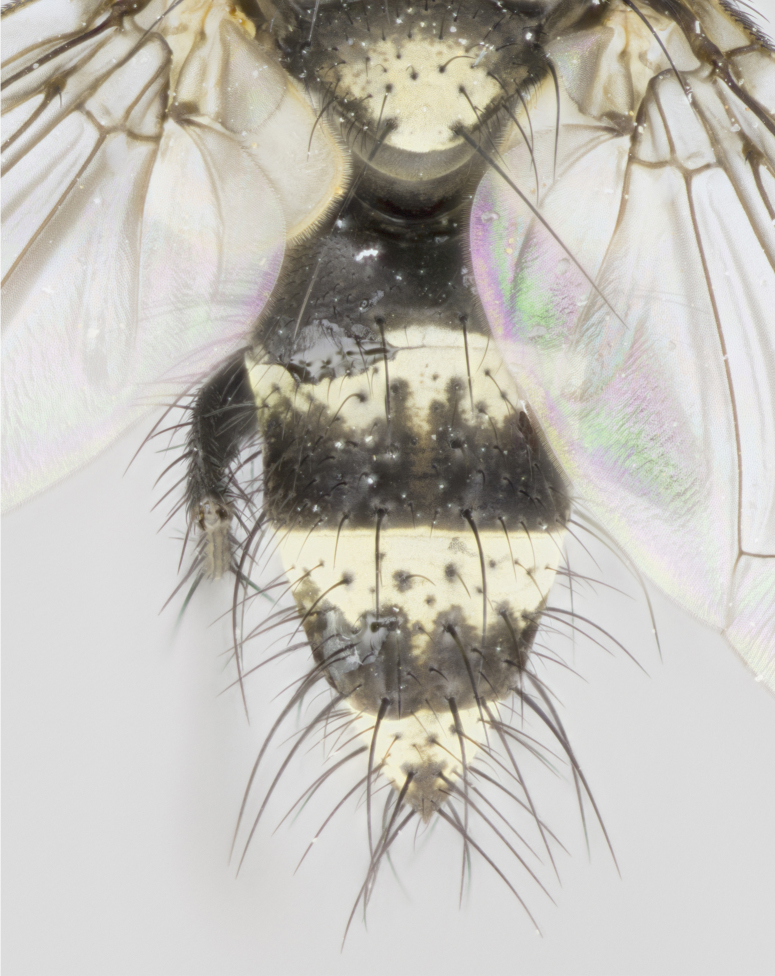
*T. panamensis*

**Figure 1c.**
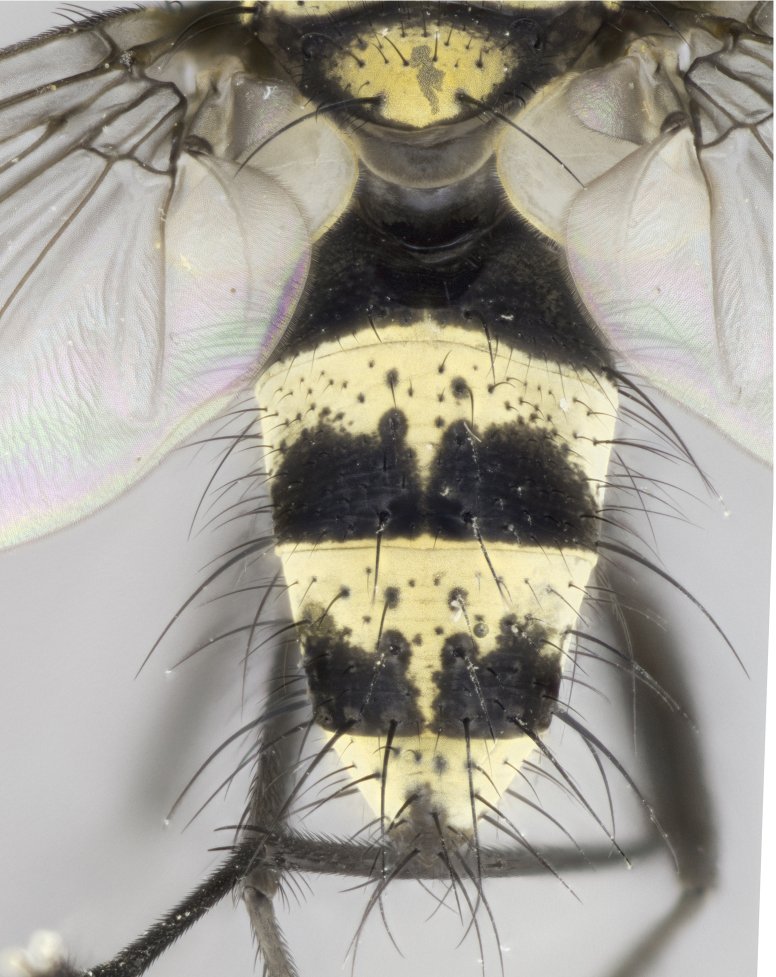
*T. josemariamoragai* **sp. n.**

**Figure 1d.**
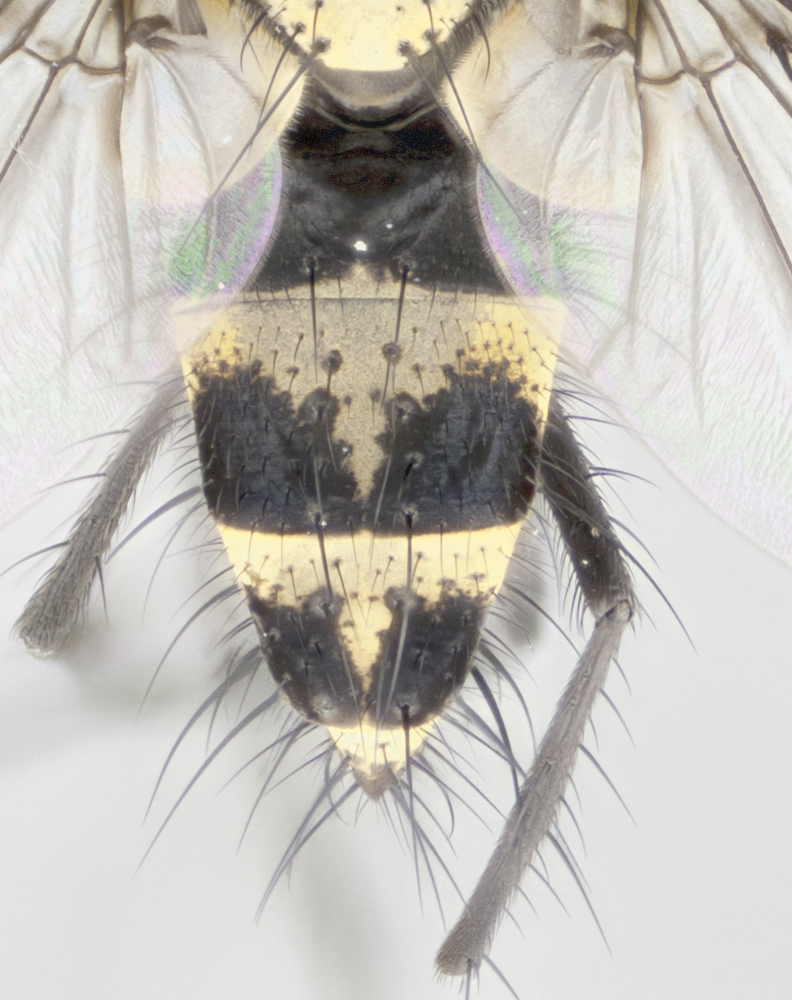
*T. edwinbermudezi* **sp. n.**

**Figure 2a.**
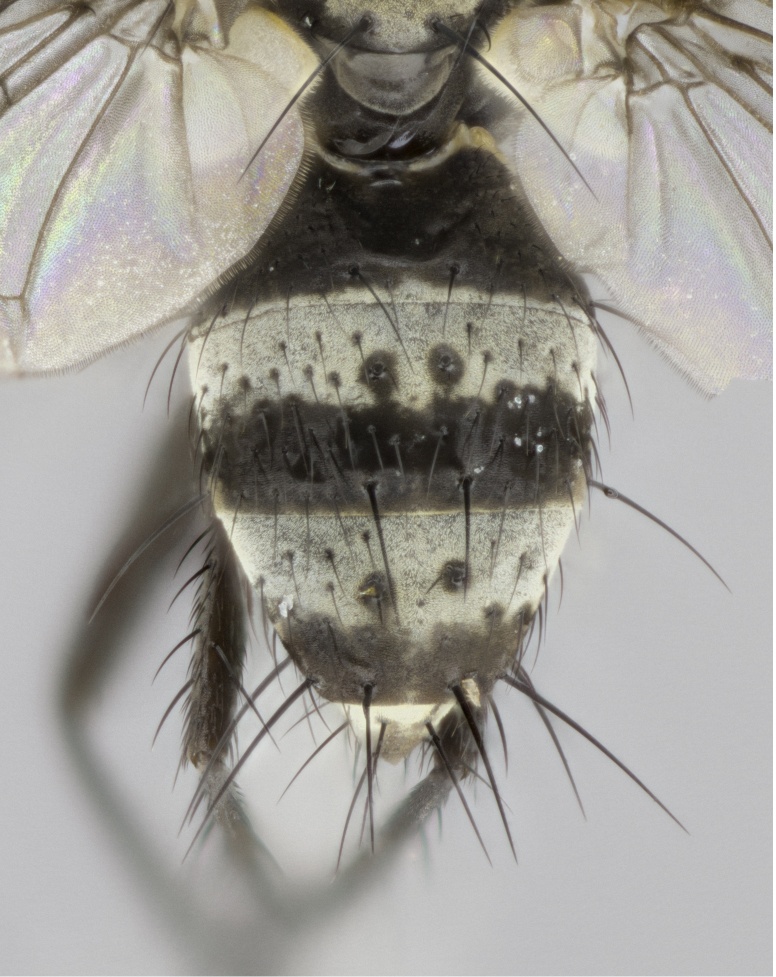
*T. uniformis* **sp. n.**

**Figure 2b.**
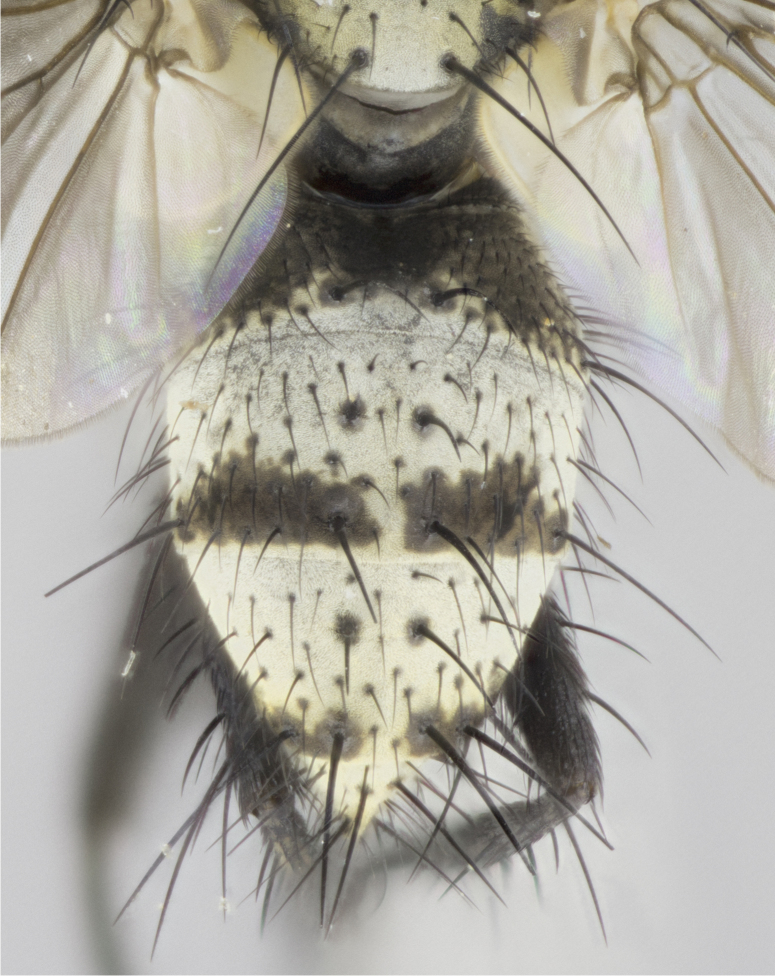
*T. panamensis*

**Figure 2c.**
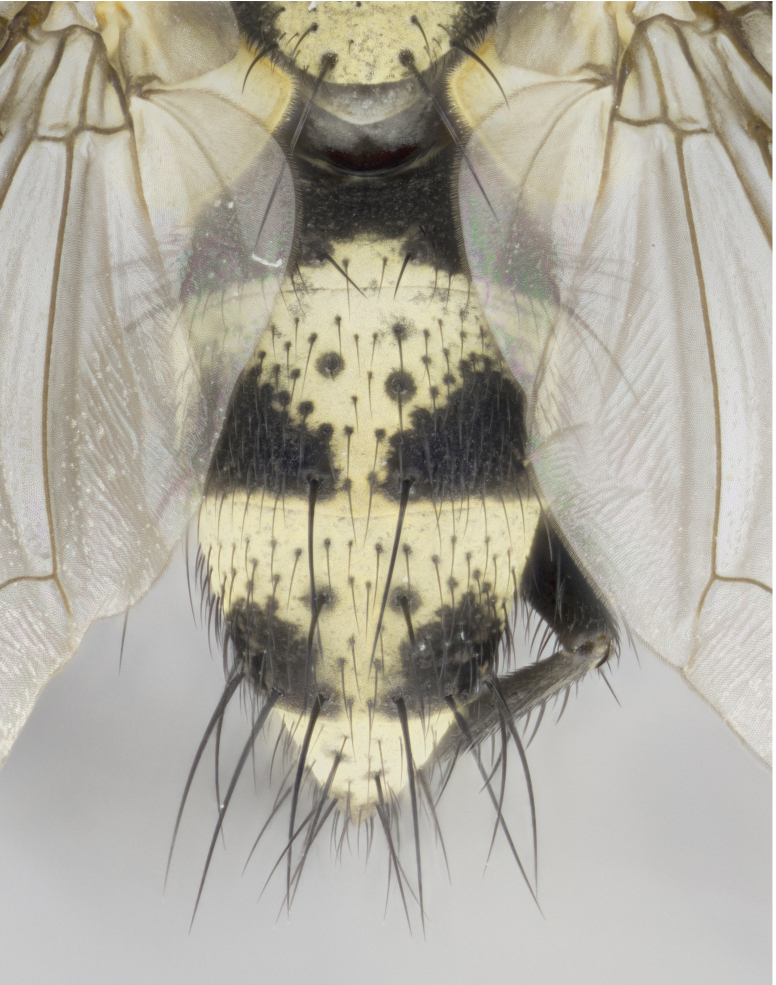
*T. edwinbermudezi* **sp. n.**

**Figure 2d.**
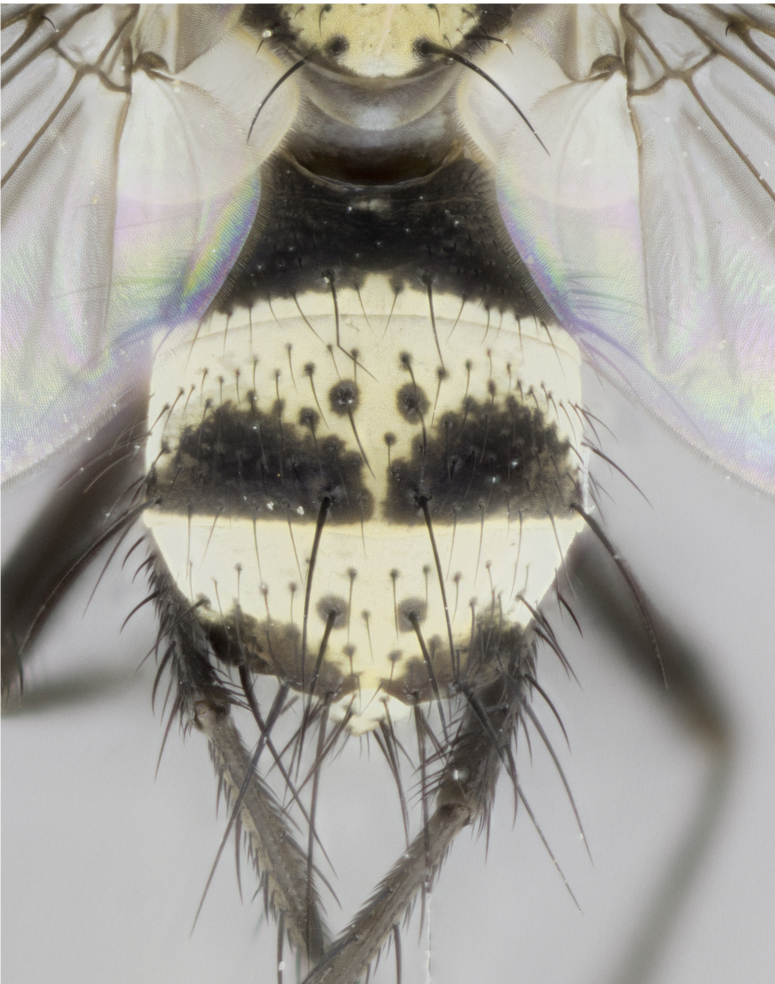
*T. josemariamoragai* **sp. n.**

**Figure 3a.**
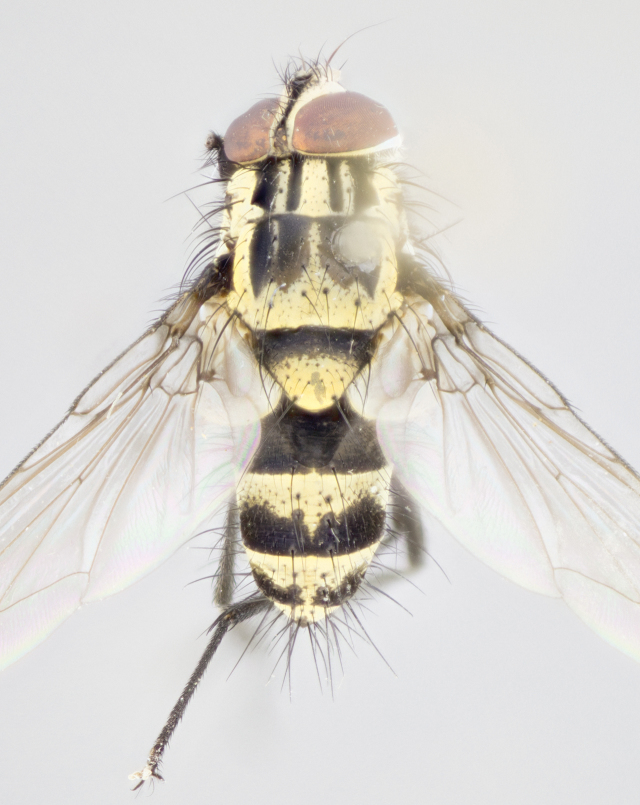
male dorsal

**Figure 3b.**
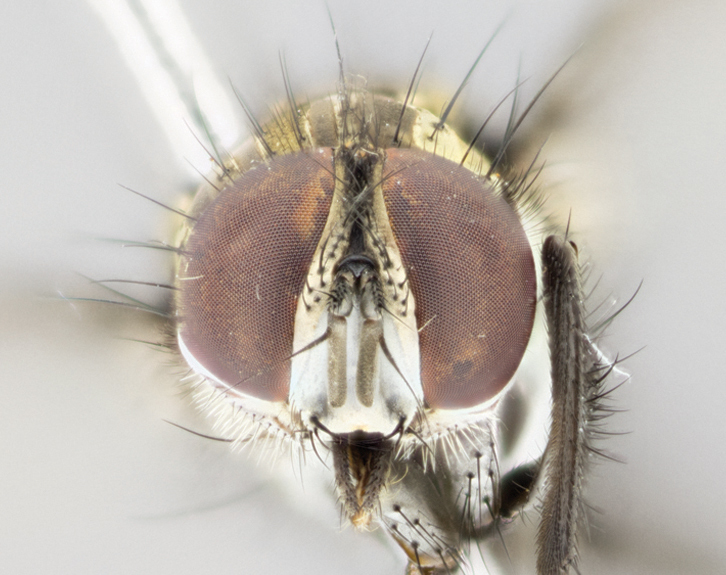
male frontal

**Figure 3c.**
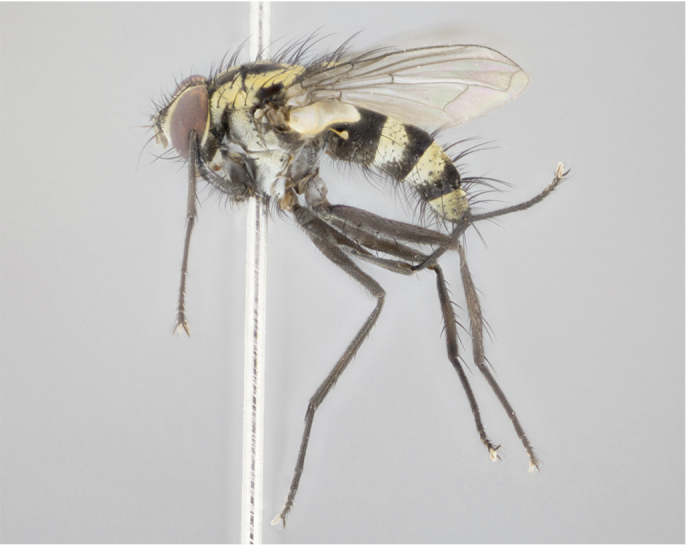
male lateral

**Figure 3d.**
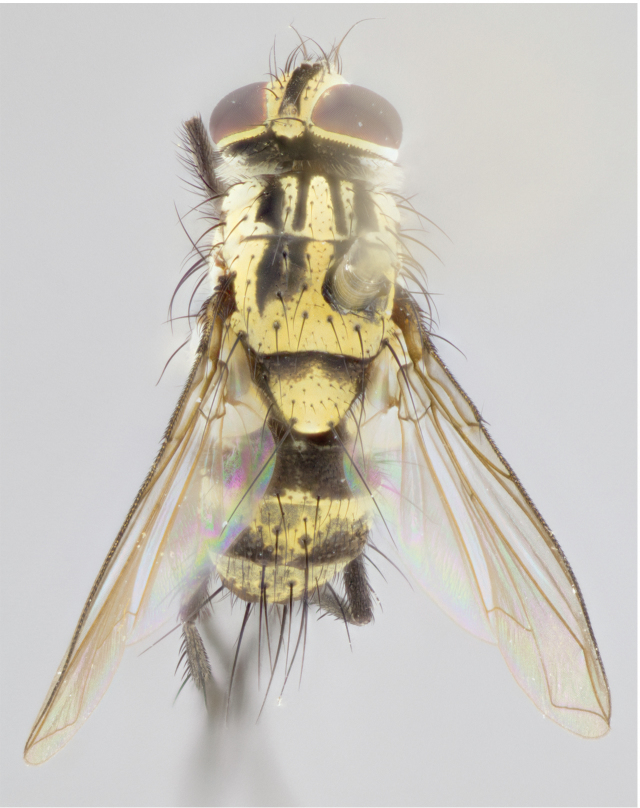
female dorsal

**Figure 3e.**
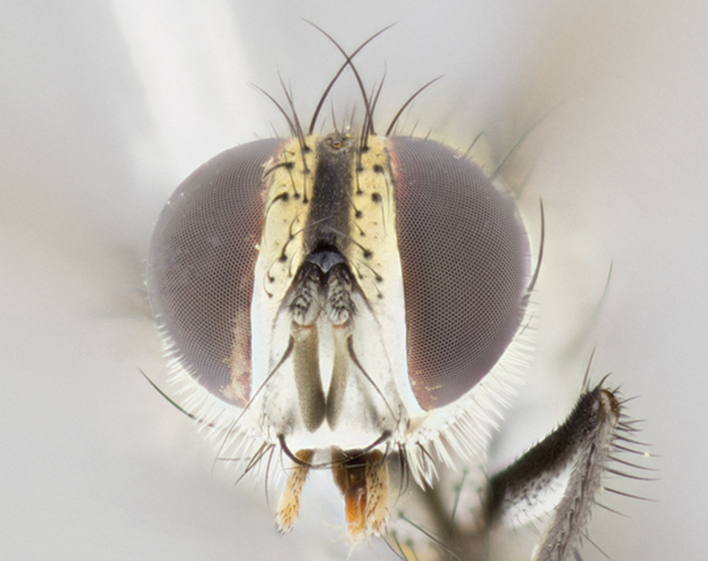
female frontal

**Figure 3f.**
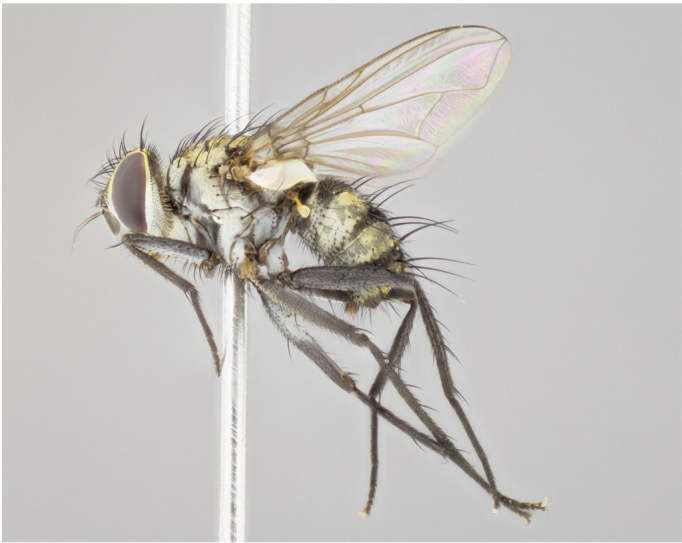
female lateral

**Figure 4a.**
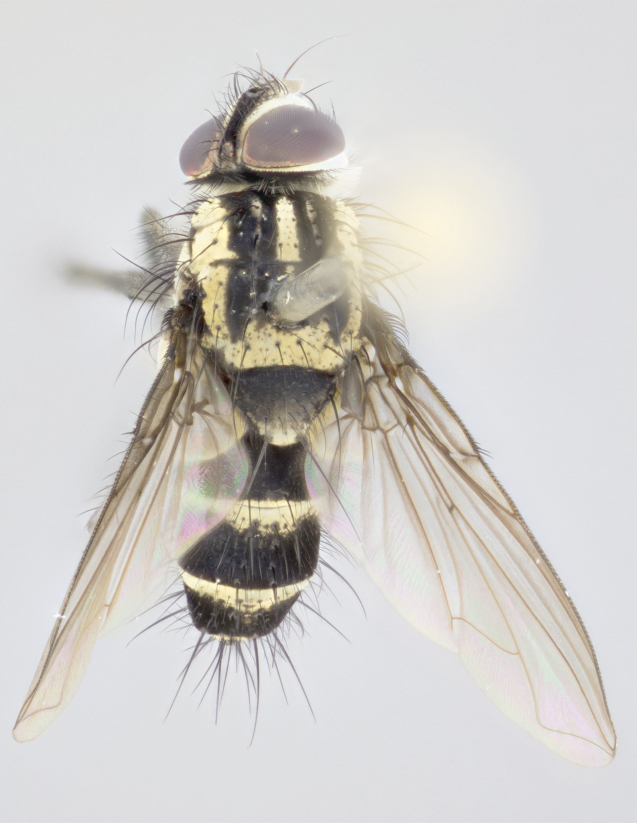
dorsal male

**Figure 4b.**
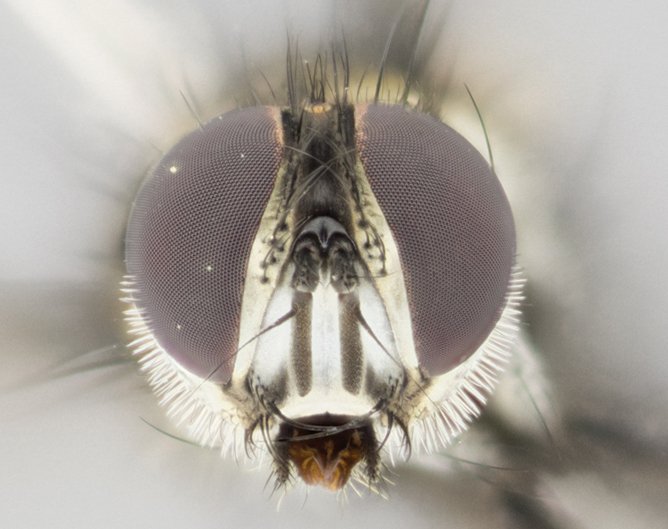
frontal male

**Figure 4c.**
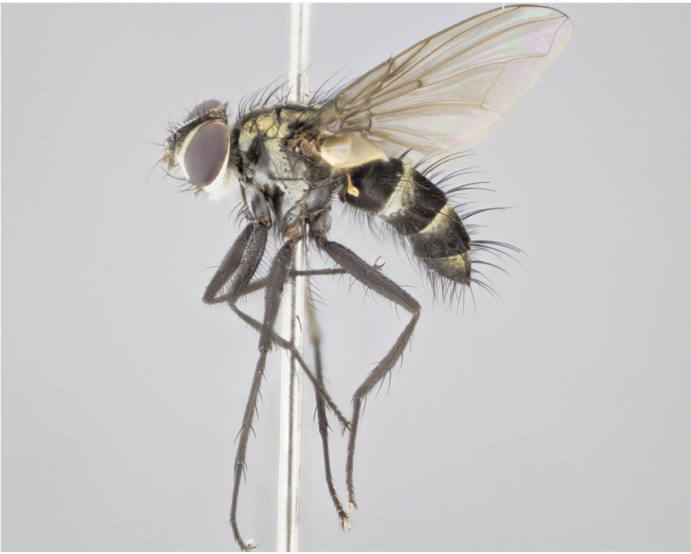
lateral male

**Figure 4d.**
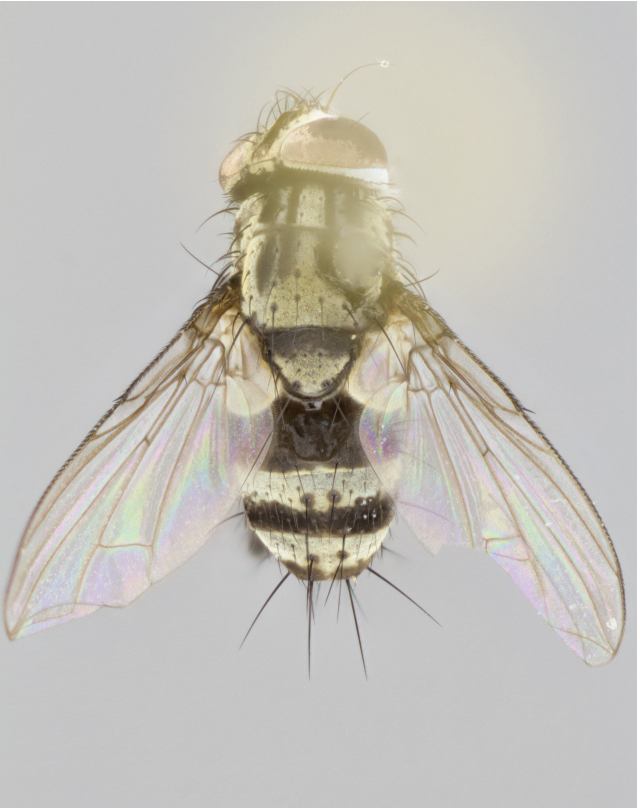
dorsal female

**Figure 4e.**
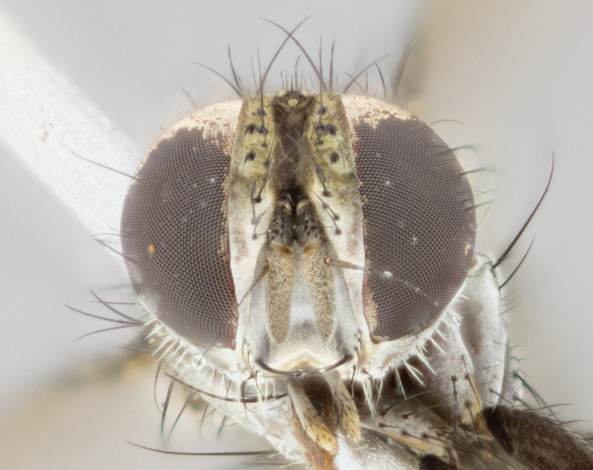
frontal female

**Figure 4f.**
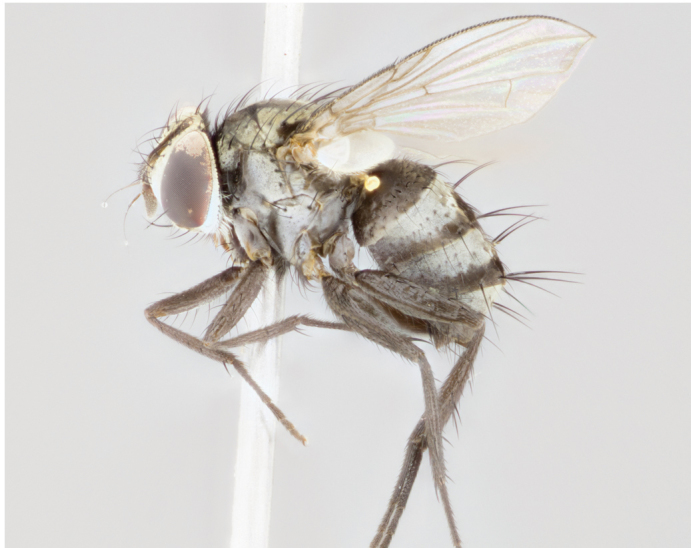
lateral female

**Figure 5a.**
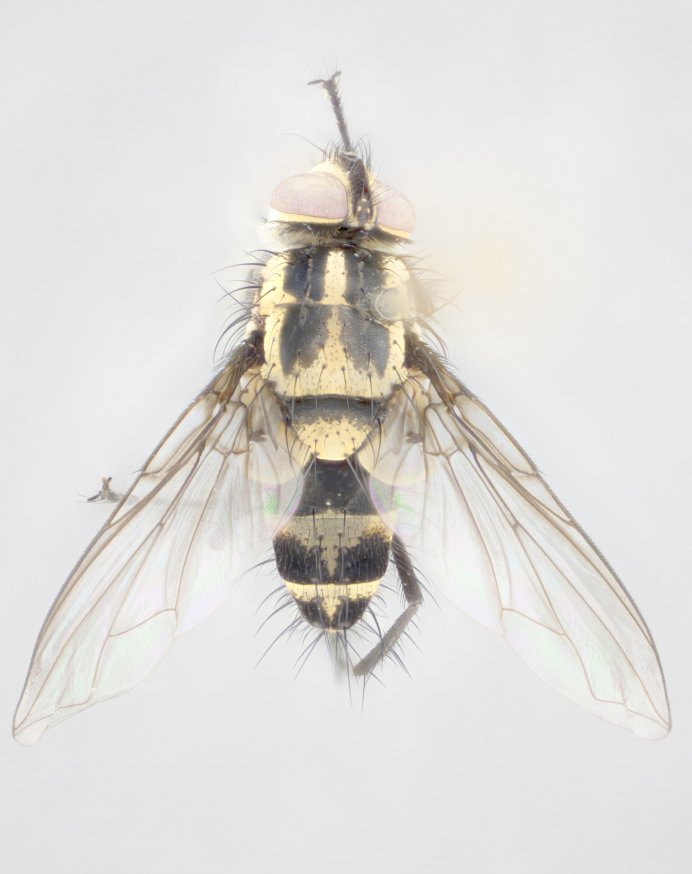
male dorsal

**Figure 5b.**
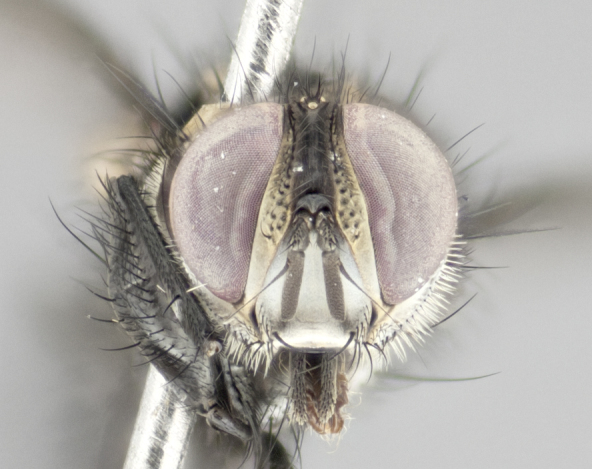
male frontal

**Figure 5c.**
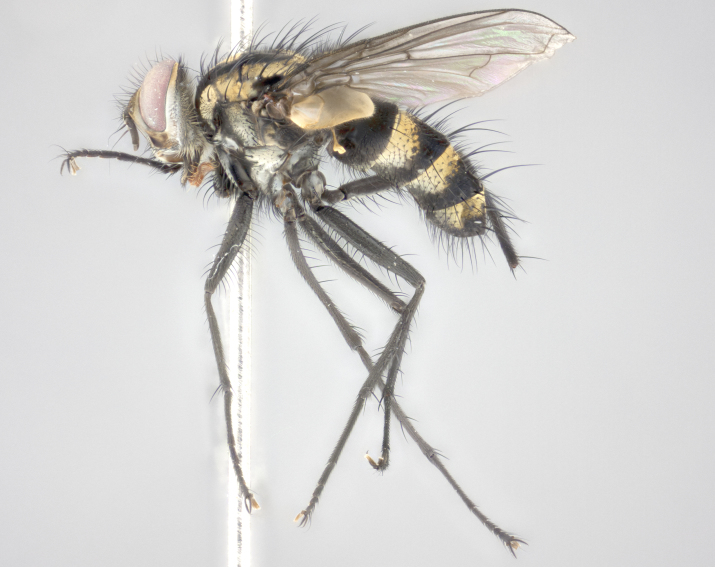
male lateral

**Figure 5d.**
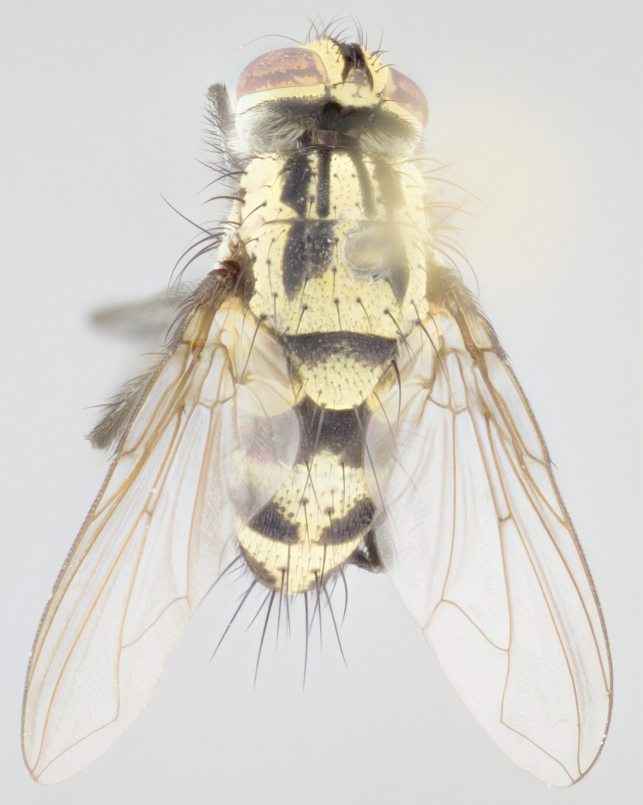
female dorsal

**Figure 5e.**
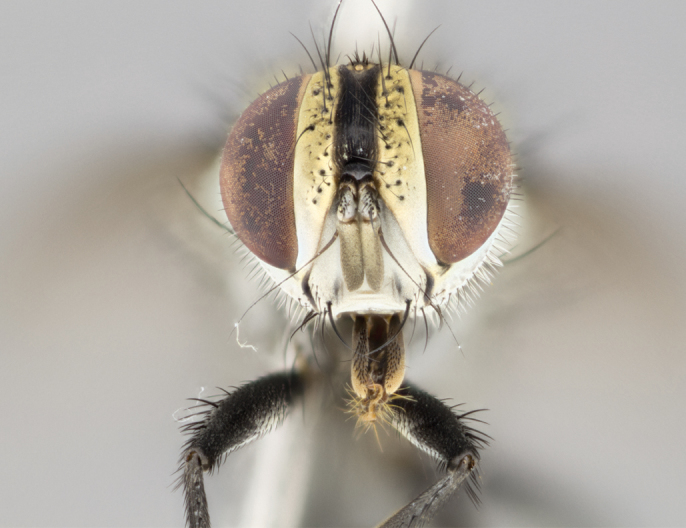
female frontal

**Figure 5f.**
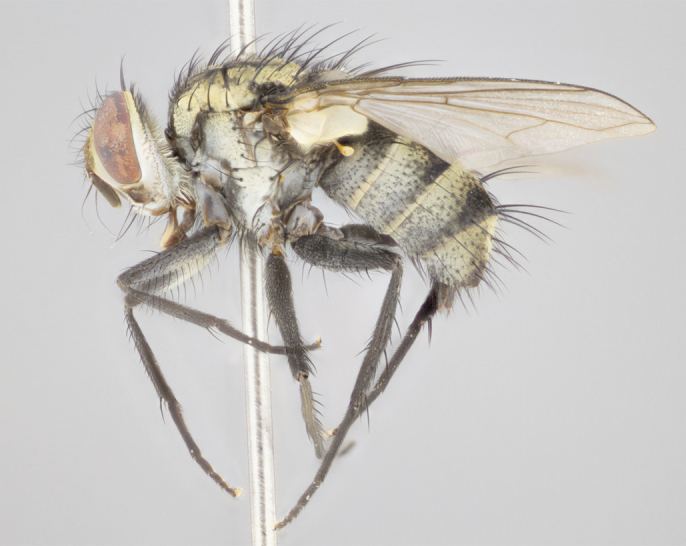
female lateral

**Figure 6a.**
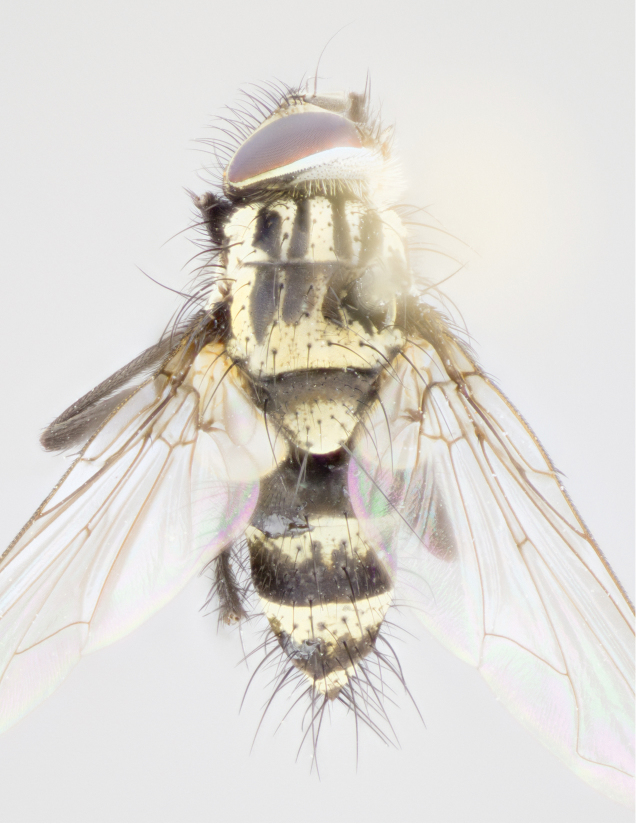
male dorsal

**Figure 6b.**
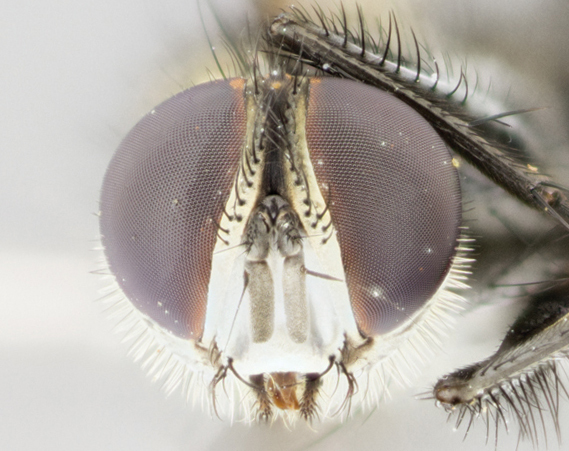
male frontal

**Figure 6c.**
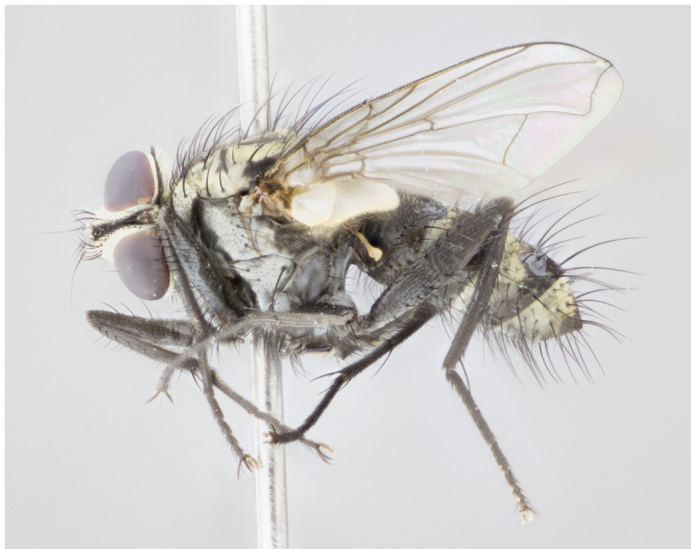
male lateral

**Figure 6d.**
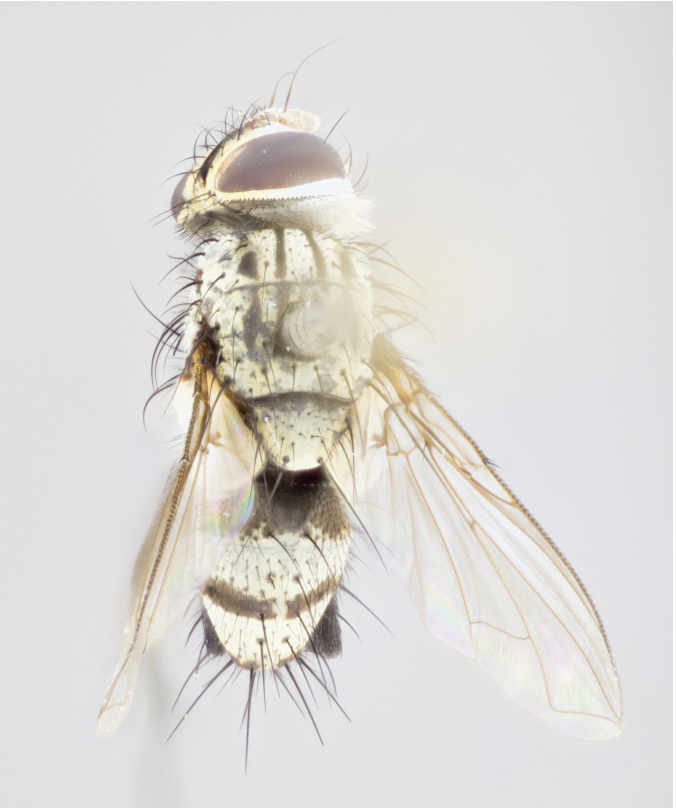
female dorsal

**Figure 6e.**
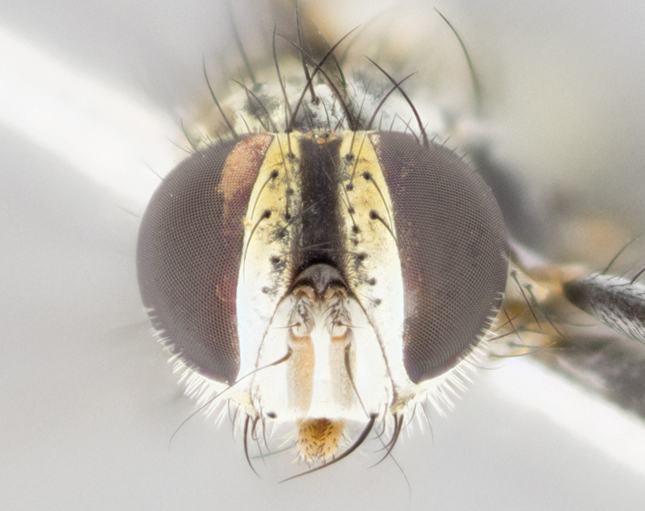
female frontal

**Figure 6f.**
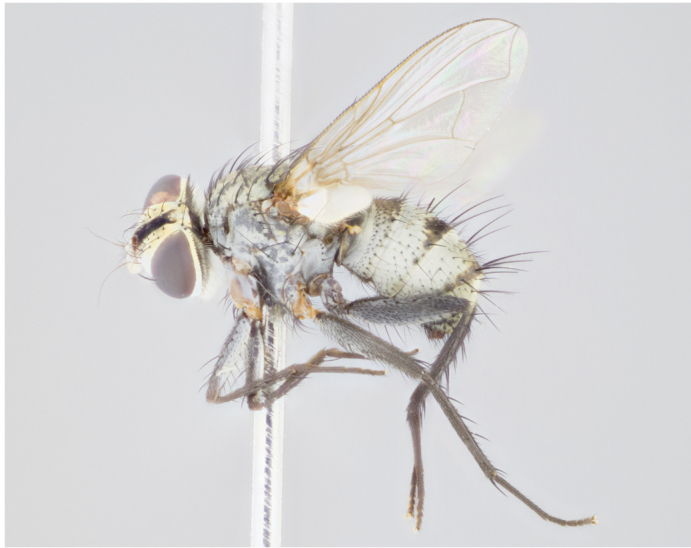
female lateral
