# Corrigendum: Three new species of *Trigonospila* Pokorny (Diptera: Tachinidae), from Area de Conservación Guanacaste, northwestern Costa Rica, with a key for their identification

**DOI:** 10.3897/BDJ.3.e6459

**Published:** 2015-10-07

**Authors:** AJ Fleming, D. Monty Wood, Daniel Janzen, Winnie Hallwachs, M. Alex Smith

**Affiliations:** ‡ Agriculture Agri-Food Canada, Ottawa, Canada; § University of Pennsylvania, Philadelphia, United States of America; | Department of Integrative Biology and the Biodiversity Institute of Ontario, Guelph, Canada

## Main Text

In preparation of the manuscript (Fleming et al. 2015) the authors incorrectly misspelled the patronym dedicated to Jose Mario Moraga. Therefore Based on Art. 32.5.1.1 of the International Code of Zoological Nomenclature (International Commission on Zoological Nomenclature 1999), and considering the etymology of the species we hereby act to correct the incorrect original misspelling of the name *Trigonospila josemariamoragai* Fleming & Wood, 2015, to the correct spelling *Trigonospila josemariomoragai* Fleming & Wood, 2015. All details pertaining to original date of publication and original pagination remain the same.
