# Supplementary material for: Three new species of Trigonospila Pokorny (Diptera: Tachinidae), from Area de Conservación Guanacaste, northwestern Costa Rica, with a key for their identification
Source: Biodivers Data J. 2015 Aug 11;(3):e4595. doi: 10.3897/BDJ.3.e4595 (PMC4563158; doi:10.3897/BDJ.3.e4595)
Supplement: Supplementary material 1 — NJ tree of ACG inventory Trigonospila [file biodiversity_data_journal-3-e4595-s001.pdf]

BOLD TaxonID Tree

Title	: Three new species of Trigonospila from ACG [DS-ASTRIGON]
Date	: 13-January-2015
Data Type	: Nucleotide
Distance Model	: Kimura 2 Parameter
Marker	: COI-5P
Codon Positions	: 1st, 2nd, 3rd
Labels	: Extra Info, SampleID, Sequence Length
Filters	: Length > 200

Sequence Count	: 31
Species count	: 4
Genus count	: 1
Family count	: 1
Unidentified	: 0

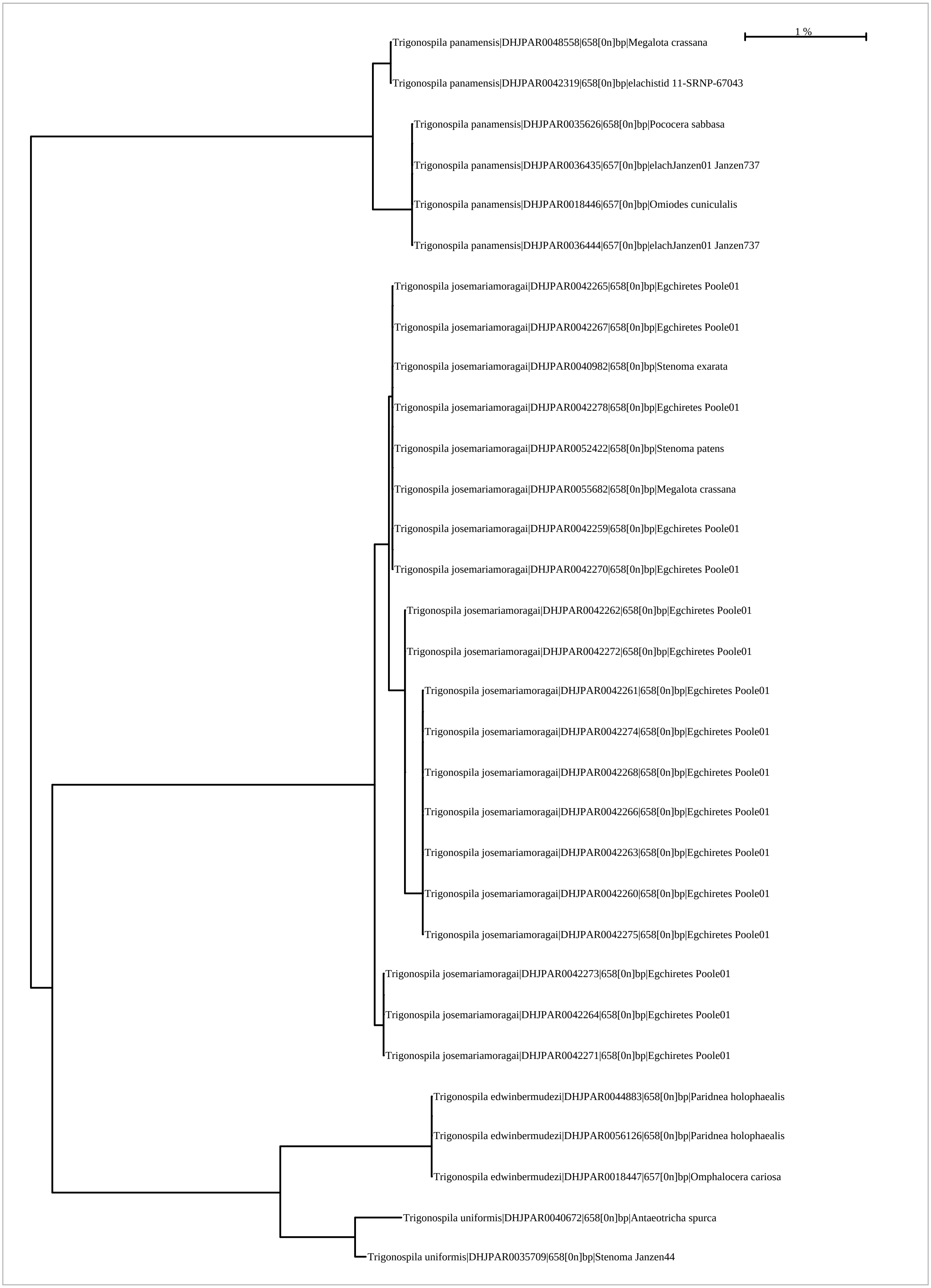
1 %
Trigonospila panamensis|DHJPAR0048558|658[0n]bp|Megalota crassana
Trigonospila panamensis|DHJPAR0042319|658[0n]bp|elachistid 11-SRNP-67043
Trigonospila panamensis|DHJPAR0035626|658[0n]bp|Pococera sabbasa
Trigonospila panamensis|DHJPAR0036435|657[0n]bp|elachJanzen01 Janzen737
Trigonospila panamensis|DHJPAR0018446|657[0n]bp|Omiodes cuniculalis
Trigonospila panamensis|DHJPAR0036444|657[0n]bp|elachJanzen01 Janzen737
Trigonospila josemariamoragai|DHJPAR0042265|658[0n]bp|Egchiretes Poole01
Trigonospila josemariamoragai|DHJPAR0042267|658[0n]bp|Egchiretes Poole01
Trigonospila josemariamoragai|DHJPAR0040982|658[0n]bp|Stenoma exarata
Trigonospila josemariamoragai|DHJPAR0042278|658[0n]bp|Egchiretes Poole01
Trigonospila josemariamoragai|DHJPAR0052422|658[0n]bp|Stenoma patens
Trigonospila josemariamoragai|DHJPAR0055682|658[0n]bp|Megalota crassana
Trigonospila josemariamoragai|DHJPAR0042259|658[0n]bp|Egchiretes Poole01
Trigonospila josemariamoragai|DHJPAR0042270|658[0n]bp|Egchiretes Poole01
Trigonospila josemariamoragai|DHJPAR0042262|658[0n]bp|Egchiretes Poole01
Trigonospila josemariamoragai|DHJPAR0042272|658[0n]bp|Egchiretes Poole01
Trigonospila josemariamoragai|DHJPAR0042261|658[0n]bp|Egchiretes Poole01
Trigonospila josemariamoragai|DHJPAR0042274|658[0n]bp|Egchiretes Poole01
Trigonospila josemariamoragai|DHJPAR0042268|658[0n]bp|Egchiretes Poole01
Trigonospila josemariamoragai|DHJPAR0042266|658[0n]bp|Egchiretes Poole01
Trigonospila josemariamoragai|DHJPAR0042263|658[0n]bp|Egchiretes Poole01
Trigonospila josemariamoragai|DHJPAR0042260|658[0n]bp|Egchiretes Poole01
Trigonospila josemariamoragai|DHJPAR0042275|658[0n]bp|Egchiretes Poole01
Trigonospila josemariamoragai|DHJPAR0042273|658[0n]bp|Egchiretes Poole01
Trigonospila josemariamoragai|DHJPAR0042264|658[0n]bp|Egchiretes Poole01
Trigonospila josemariamoragai|DHJPAR0042271|658[0n]bp|Egchiretes Poole01
Trigonospila edwinbermudezi|DHJPAR0044883|658[0n]bp|Paridnea holophaealis
Trigonospila edwinbermudezi|DHJPAR0056126|658[0n]bp|Paridnea holophaealis
Trigonospila edwinbermudezi|DHJPAR0018447|657[0n]bp|Omphalocera cariosa
Trigonospila uniformis|DHJPAR0040672|658[0n]bp|Antaeotricha spurca
Trigonospila uniformis|DHJPAR0035709|658[0n]bp|Stenoma Janzen44
